# The role of age in the relationship between brain structure and cognition: moderator or confound?

**DOI:** 10.1093/cercor/bhag024

**Published:** 2026-03-11

**Authors:** Ben Griffin, Chetan Gohil, Mark W Woolrich, Stephen M Smith, Diego Vidaurre

**Affiliations:** Oxford Centre for Functional MRI of the Brain (FMRIB), Oxford Centre for Integrative Neuroimaging, Nuffield Department of Clinical Neurosciences, University of Oxford, John Radcliffe Hospital, Headley Way, Headington, Oxford, Oxfordshire OX3 9DU, United Kingdom; Center of Functionally Integrative Neuroscience, Department of Clinical Medicine, Aarhus University, Building 1710, Universitetsbyen 3, 8000 Aarhus, Denmark; Oxford Centre for Human Brain Activity (OHBA), Oxford Centre for Integrative Neuroimaging, Department of Psychiatry, University of Oxford, Warneford Hospital, Warneford Lane, Headington, Oxford, Oxfordshire OX3 7JX, United Kingdom; Oxford Centre for Human Brain Activity (OHBA), Oxford Centre for Integrative Neuroimaging, Department of Psychiatry, University of Oxford, Warneford Hospital, Warneford Lane, Headington, Oxford, Oxfordshire OX3 7JX, United Kingdom; Oxford Centre for Human Brain Activity (OHBA), Oxford Centre for Integrative Neuroimaging, Department of Psychiatry, University of Oxford, Warneford Hospital, Warneford Lane, Headington, Oxford, Oxfordshire OX3 7JX, United Kingdom; Oxford Centre for Functional MRI of the Brain (FMRIB), Oxford Centre for Integrative Neuroimaging, Nuffield Department of Clinical Neurosciences, University of Oxford, John Radcliffe Hospital, Headley Way, Headington, Oxford, Oxfordshire OX3 9DU, United Kingdom; Center of Functionally Integrative Neuroscience, Department of Clinical Medicine, Aarhus University, Building 1710, Universitetsbyen 3, 8000 Aarhus, Denmark; Oxford Centre for Human Brain Activity (OHBA), Oxford Centre for Integrative Neuroimaging, Department of Psychiatry, University of Oxford, Warneford Hospital, Warneford Lane, Headington, Oxford, Oxfordshire OX3 7JX, United Kingdom; Centre de Recerca Matemàtica, Campus UAB, Carrer de l'Albareda, Edifici C, 08193 Bellaterra, Barcelona, Spain

**Keywords:** elastic net regression, individual differences, lifespan neuroscience, moderation analysis, structural MRI

## Abstract

Understanding how differences in brain structure relate to differences in cognition across the lifespan is essential for addressing age-related cognitive decline. Since age is strongly associated with both brain structure and cognition, predictive models often risk simply capturing age effects. To mitigate this risk, deconfounding is typically applied to remove the effects of age. Here, beyond treating age as a confound, we treat it as a moderator by estimating brain-cognition associations separately across age groups. This captures age-stratified changes in how brain structure and cognitive performance are statistically connected. For this view to hold, variations in brain structure linked to differences in cognitive performance in older subjects (eg related to disease) would differ from those in younger subjects. Using structural brain imaging data from the UK Biobank we found an asymmetry in generalisability: models trained on younger subjects successfully predicted cognition in older subjects, but models trained on older subjects failed to generalize to younger individuals. These findings reveal a trade-off between model specificity and generalisability, suggesting the optimal approach—whether age-specific or pooled—depends on the research or clinical goal for the target population.

## Introduction

Identifying robust associations between brain structure and cognition is critical for both basic neuroscience and clinical applications. Predictive models that reveal relationships between brain data (eg structural magnetic resonance imaging (MRI)) and cognitive abilities (eg fluid intelligence) are important for uncovering these associations, improving our understanding of cognitive decline and neurological disorders such as Alzheimer’s disease (Solé-Padullés et al. 2009) and other dementias (Suzuki et al. 2019).

Capturing these brain-cognition relationships is complicated by the fact that aging affects both brain structure and cognition in complex ways that vary across individuals. For instance, different cognitive abilities show different relationships with age (Horn and Cattell 1967); crystallized abilities generally improve until 60 before plateauing, whereas fluid abilities begin to decline from age 20 (Salthouse 2010; Murman 2015). Similarly, different brain regions show distinct patterns of age-related atrophy (Fjell and Walhovd 2010; Bethlehem et al. 2022). The complexity increases when considering structure and cognition together. For example, dividing adults aged 18 to 88 into younger and older groups revealed a weaker relationship between white matter and certain cognitive functions in older adults, suggesting that some brain-cognition associations become less stable with age (de Mooij et al. 2018).

These complexities make robust predictive modeling challenging, particularly when accounting for age. Although age is a key variable in certain studies of age-related conditions (Davis et al. 2018; Jack et al. 2019), it is often treated as a confound, and typically linearly regressed out from both the brain data and cognitive variables (Bracher-Smith et al. 2022; Azevedo et al. 2023). This approach ensures that identified relationships are truly reflective of brain-cognition associations independent of age, at least under assumptions of linearity. Nonetheless, models can still show age-related bias. While such biases are well documented for sex, ethnicity, and socio-economic status (Martin et al. 2019; Obermeyer et al. 2019; Li et al. 2022), they also arise for age (Greene et al. 2022; Wu et al. 2023). This is consistent with evidence that predictive models can also show limited generalisability across behavioral domains (eg cognition vs. personality; (Chen et al. 2022). These complexities highlight the importance of developing predictive approaches that perform robustly across age groups and call into question whether age should be treated merely as a nuisance variable to be removed.

Taken together, these considerations raise a central question: do the brain features linked to better cognition differ between younger and older adults, potentially shifting toward aging trajectories and atrophy in later life, and does this limit cross-age generalisability? To address this, we move beyond treating age solely as a linear confound and also consider its role as a moderator of brain-cognition relationships. Here, we use “moderation” in a broader sense to refer to age-dependent differences in brain-cognition associations, rather than the more orthodox estimation of a single continuous age × imaging-derived phenotype (IDP) interaction term (Burzynska et al. 2012; de Chastelaine et al. 2019; de Chastelaine et al. 2023). While moderation is traditionally tested using continuous interaction terms, such approaches can fail to distinguish between true interaction effects and underlying nonlinear age relationships (Rimpler et al. 2024).

We characterize age-dependent differences using age-stratified univariate analyses of structural IDPs, which allow associations to vary flexibly across age groups. We then use cross-validated multivariate prediction to test how well brain-cognition mappings learned in one age group generalize to others. Given that aging involves different underlying mechanisms (eg neurodegeneration), we hypothesize that the structural brain features associated with cognitive performance in older adults may differ from those in younger populations.

Using data from UK Biobank (UKB) (Sudlow et al. 2015), we show age-dependent differences in how predictive models generalize across age. While certain brain-cognition associations appear stable across the lifespan, we reveal an asymmetry: models trained on younger subjects generalize well to older subjects, but models trained on older subjects fail to capture the relationships relevant to a younger population. This suggests that while certain brain-cognition associations are stable across the lifespan, there may be increased noise, variability, or complexity in the relationships for older subjects that impact model generalisability. These results highlight a trade-off between specificity and generalisability, with important implications for both research and clinical applications.

## Materials and methods

### UK Biobank neuroimaging and non-imaging data

We analyzed data from UKB, a large-scale neuroimaging dataset that includes subjects aged 47 to 83 years. Our study explored the relationship between a composite cognitive measure (described below) and 1439 structural IDPs. These IDPs comprised 1436 T1-weighted MRI measures, including Regional and Tissue Volume, Cortical Area, Cortical Thickness, Regional and Tissue Intensity, Cortical Gray-White Contrast, and White Matter Hyperintensity Volumes, along with three T2-weighted MRI measures.

Our analysis included 25,170 UKB subjects, after excluding those with missing values for age or any of the cognitive traits used to construct the composite measure. Structural IDPs were obtained from the UKB imaging pipeline, which applies standard preprocessing and quality control procedures prior to data release (Alfaro-Almagro et al. 2018). No additional study-specific exclusions based on MRI quality or health-related criteria were applied. Missing values in structural IDPs were imputed using the mean to maintain a larger sample size. Overall missingness was low (2.17% of IDP entries across 1439 IDPs), with no strong age-related differences; detailed diagnostics, including missingness by IDP category and age group, are reported in Fig. S1.

Age was used to define analysis-specific groupings. For analyses examining finer age-dependent variation, subjects were stratified into quartiles, as used in the univariate modeling (Fig. S2 shows the age distribution and quartile cut-points). For the multivariate analyses, we split the cohort into younger and older halves at the median age to maximize sample size per group and reduce overfitting risk in high-dimensional models. Figure S3 shows this split and the corresponding sex distribution, confirming similar male–female proportions within each age group.

All participants provided informed consent, and UK Biobank obtained ethical approval from the National Health Service National Research Ethics Service (Ref 11/NW/0382). This study was conducted under UK Biobank application number 8107.

#### Confound removal

Large-scale neuroimaging datasets such as UKB have significantly increased the statistical power for detecting small brain-behavior relationships but have also increased the risk of confounding biases (eg age, head motion), which may bias findings and complicate interpretation (Smith and Nichols 2018). To control for confounding effects, we linearly regressed out the confounds from both imaging and cognitive variables.

We created a reduced set of confounds from a comprehensive set of 562 UKB imaging confounds as described in (Alfaro-Almagro et al. 2021; Table S1). First, we selected 17 essential confounds including age, sex, age × sex, scanning site, head size, and head motion (Table S2). These essential confounds were treated as explicit confound regressors and were excluded from dimensionality reduction.

We then summarized the remaining 545 confounds (including additional age-related terms such as age^2^) using Principal Component Analysis (PCA). PCA captured shared variance across correlated confounds in a smaller set of orthogonal components, avoiding redundancy and multicollinearity. We retained the principal components that together explained >85% of the total variance, consistent with previous work using UKB confounds (Farahibozorg et al. 2021).

When PCA was fit once on the full dataset (as in the univariate analyses), retaining components explaining >85% of variance corresponded to 211 components. In the multivariate cross-validation analyses, PCA was fit within each training fold and applied to held-out data; the number of retained components was highly stable across folds (median 197, range 196 to 197). This captured most of the variability of the full confound set in a lower-dimensional form while avoiding instability from including hundreds of highly correlated variables directly. Deconfounding was performed prior to modeling and, for cross-validation analyses, within training folds with transformations applied to held-out data to prevent leakage. The same confound specification (17 essential confounds plus PCA-reduced nuisance components) was used across analyses; only the fitting procedure differed (full-dataset fit for univariate analyses versus within-fold fitting for cross-validated analyses).

Age-related terms were included as confounds to prevent models from exploiting shared age dependence (ie IDPs acting as proxies for chronological age). This deconfounding removes *direct* age effects but still allows the residual IDP-cognition association to vary across age groups, which we assess using age-stratified analyses and cross-age generalization tests. As a diagnostic, we quantified the additional variance explained by age-related terms beyond non-age confounds (Fig. S4 and Table S3).

#### Composite cognitive measure

Prediction accuracy for individual cognitive traits is often limited (Pervaiz et al. 2020), and dividing the sample into age groups further reduces statistical power. To obtain a stable and interpretable cognitive target, we therefore constructed a composite measure from multiple UKB cognitive traits.

Briefly, cognitive traits were first screened for sufficient data coverage and ranked by their brain-cognition predictability using preliminary elastic net models fitted across all subjects. Based on this screening, the top 30 traits were selected and combined using PCA, with the first principal component used as the final cognitive target. Trait screening was performed once to define a consistent outcome variable and was independent of age stratification analyses. Full methodological details and diagnostic analyses motivating these choices are provided in Methods S1 and Fig. S5. The robustness of the cognitive composite to alternative construction strategies was assessed using sensitivity analyses reported in Fig. S6.

### Univariate modeling: age-moderated effects

To investigate whether associations between brain structure and cognitive performance vary across the lifespan, we applied univariate linear models to estimate age-dependent effects of individual IDPs. This section outlines the modeling approach and describes the permutation testing framework used to assess the statistical significance of the effects.

#### Linear modeling of age-dependent brain-cognition relationships

All IDPs and cognitive measures were first deconfounded to remove confounding variance, including direct age-related effects. Importantly, this procedure removes variance explained by age itself, but does not constrain how the residual IDP-cognition association may differ across age-defined subgroups. As the univariate analyses did not involve a trait-test split, deconfounding was applied once across all subjects prior to age stratification.

To estimate age-specific brain-cognition associations, subjects were stratified into four age quartiles (Q1-Q4). Age quartiles were used to provide a simple, interpretable, and robust characterization of age-dependent effects that does not rely on strong parametric assumptions about the functional form of age dependence. For each IDP, we constructed four quartile-specific regressors by multiplying the IDP by binary indicators of age quartile membership. This yielded four coefficients per IDP, each representing the association between that IDP and cognition within a specific age quartile.

IDPs were standardized (z-scored) within each age quartile after deconfounding, ensuring comparability of coefficient magnitudes across quartiles. No additional confound regressors were included in the univariate models, as confounding effects had already been removed from both imaging and cognitive variables prior to analysis. Each model therefore consisted of quartile-specific intercepts (implemented as age-quartile indicator variables) and four quartile-specific IDP regressors. This allows baseline cognition to differ across quartiles, ensuring slope differences reflect within-quartile associations rather than between-quartile mean shifts. Under this formulation, quartile-specific coefficients quantify brain-cognition associations after removing direct age-related variance, and differences across quartiles reflect age-dependent differences in association rather than residual age confounding (Rao et al. 2015).

#### Permutation testing of age-moderated effects

To test whether IDP-cognition associations differed across age quartiles, we used permutation testing on the age-stratified regression coefficients. Specifically, the regression model yielded four IDP coefficients (one for each age quartile). For each quartile, we computed the difference between the coefficient for each quartile and the mean of the other three (test statistic and procedure in Methods S2).

We generated null distributions by randomly permuting subjects’ age-quartile assignments 10,000 times and recomputing these coefficient-difference statistics (ie the quartile-versus-others contrasts). Permutation *P*-values were computed using a + 1 correction, yielding four *P*-values per IDP (one per quartile). Statistical significance was assessed using Benjamini-Hochberg false discovery rate (FDR) correction across all IDP × quartile tests.

In addition to testing individual IDPs, we assessed population-level evidence for age-dependent effects using global enrichment analyses of the permutation *P*-values. Specifically, we tested for non-uniformity using a Kolmogorov–Smirnov test, estimated the proportion of null associations using Storey’s π₀, and combined evidence across IDPs using a permutation-based Fisher test (Methods S3). These complementary analyses provide sensitivity to weak, distributed effects that may not survive stringent multiple-comparison correction at the single-IDP level (Fisher 1970; Nichols and Holmes 2002; Storey 2002).

### Multivariate modeling: elastic net prediction

**Figure 1:**
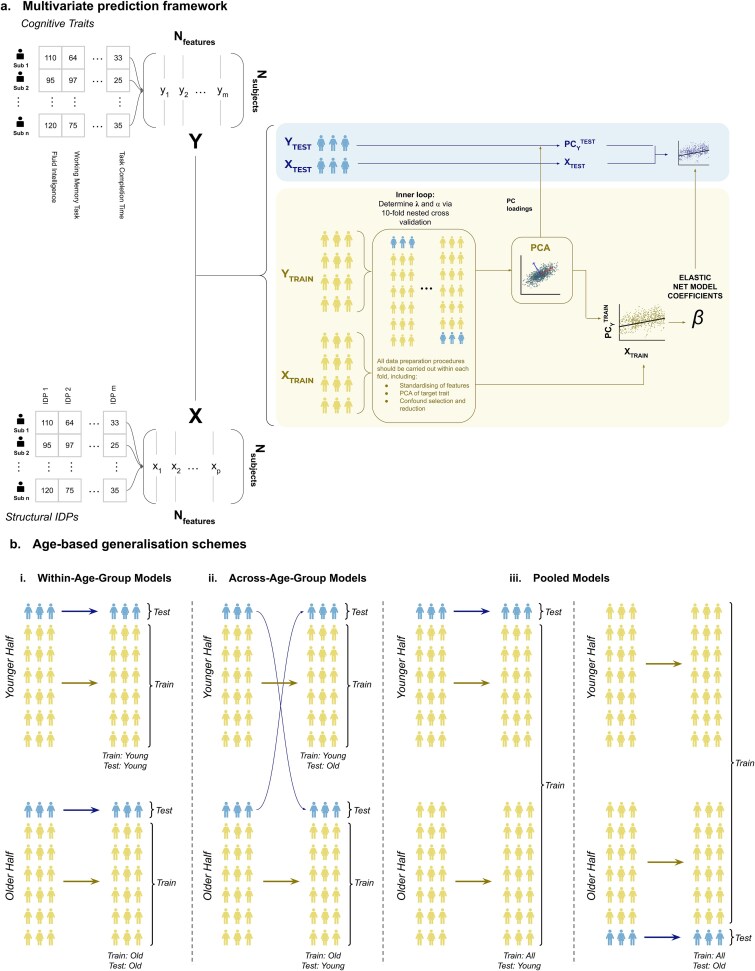
Multivariate prediction framework and age-based generalization schemes. a) Multivariate prediction framework. Schematic of the nested cross-validation framework used for multivariate prediction of cognition from structural IDPs. Within each training fold, all data preparation steps, including standardization, confound removal, and construction of the composite cognitive target via principal component analysis (PCA), were performed using training data only to avoid information leakage. The learned PCA loadings and elastic net model coefficients were then applied to held-out test data to generate out-of-sample predictions. b) Age-based generalization schemes. Training and testing strategies used to assess model generalization across age groups: **(i) within-age-group models**, trained and tested within the same age group; **(ii) across-age-group models**, trained on one age group and tested on the other; and **(iii) pooled models**, trained on subjects spanning the full age range (subsampled to match training set sizes) and tested within a specific age group. The schematic illustrates a single fold of the cross-validation procedure. Across all approaches, models were trained and evaluated on equal numbers of subjects per fold.

This section describes the multivariate prediction framework used to assess model generalization across different age groups (Fig. 1a). To prevent information leakage and ensure comparability across age groups and training regimes, all data preparation steps in the multivariate analyses were performed within cross-validation folds. This included deconfounding of imaging and cognitive variables, feature standardization, and construction of the composite cognitive target via PCA, with all transformations estimated on the training data and applied unchanged to the held-out test data, irrespective of whether models were trained or tested on younger, older, or pooled subjects (Snoek et al. 2019).

We used elastic net regression to predict cognition from all 1439 structural IDPs simultaneously. Unlike the univariate analyses, which examined IDPs one at a time, this approach incorporates all structural features together, allowing the model to capture brain-wide patterns associated with cognition and improving predictive performance in a way that aligns more closely with real-world or clinical applications.

#### Multivariate prediction using elastic net regression

Elastic net is a common approach for handling multicollinearity in large imaging datasets while avoiding overfitting. Elastic net combines the L₁ penalty of lasso (Tibshirani 1996) and the L₂ penalty of ridge regression (Hoerl and Kennard 1970), enabling both coefficient shrinkage and sparsity in the model (Zou and Hastie 2005; Vidaurre et al. 2013).

Let $X\in{\mathbb{R}}^{n\times p}$ be the design matrix with $n$ subjects and $p$ features, and let $y\in{\mathbb{R}}^n$ be the target vector, representing the composite cognitive measure. Following the implementation in scikit-learn, the elastic net regression coefficients, $\hat{\beta}$, are determined by:

$\hat{\beta}=\underset{\alpha }{\mathrm{argmin}}\left\{\frac{1}{2n}\ {\left|\left|y- X\beta \right|\right|}_2^2+\alpha \left[\rho{\left|\left|\beta \right|\right|}_1+\frac{1-\rho }{2}{\left|\left|\beta \right|\right|}_2^2\right]\right\}.$

Here, $\alpha$ is the overall regularization strength, and $\rho$ (equivalent to l1_ratio in scikit-learn) controls the mix between L1 and L2 penalties. Both hyperparameters were selected via nested cross-validation, and all IDPs were z-scored within each training fold (scaling applied to test folds) to ensure comparability across predictors.

#### Age-split training and generalization performance

Using a fully cross-validated framework, we applied elastic net regression with 1439 IDPs to predict cognition, dividing the population into two equally sized age groups (younger and older halves). We opted for two groups, rather than four as in the univariate analyses, to simplify the analysis and ensure larger sample sizes per group. This grouping improved model stability and statistical power during cross-validation, while still capturing meaningful age differences. Prior to modeling, all confounds (including age and age-related terms such as age^2^) were regressed out from both predictors and the cognitive target, ensuring that predictions reflected brain-cognition associations independent of known confound effects, consistent with recommended best practice in large-scale neuroimaging analyses. Model performance was evaluated using 10-fold cross-validation, repeated 10 times with different random partitions of the data, resulting in 100 out-of-sample performance estimates.

The population was divided into two equally sized age groups, a younger half and an older half. We examined six distinct training and testing approaches, as shown in Fig. 1b:

1. **Within-age-group models:** Training and testing within the same age group
  Train: Young; Test: Young
  Train: Old; Test: Old
2. **Across-age-group models:** Training in one age group and testing in the other
  Train: Young; Test: Old
  Train: Old; Test: Young
3. **Pooled models:** Training on the entire dataset and testing within each specific age group
  Train: ALL; Test: Young
  Train: ALL; Test: Old

To ensure comparability across models, we matched the sample size of the Train: ALL dataset to each age-specific training set by randomly subsampling subjects. This provided a standardized framework for evaluating out-of-sample predictions across all approaches.

Differences in prediction performance between training regimes were assessed using two-sided paired Wilcoxon signed-rank tests across matched cross-validation repetitions, with Benjamini-Hochberg false discovery rate correction applied across planned pairwise contrasts. Planned contrasts included comparisons between within-age-group, pooled, and across-age-group models, as well as direct comparisons of young-trained versus old-trained models within each test group to assess asymmetries in age generalization.

#### Evaluation metrics

We report Pearson correlation (r), widely used in neuroimaging prediction studies (Vieira et al. 2022) as the primary accuracy metric, because it is invariant to differences in mean and variance and therefore suitable for assessing generalisability across age groups. In contrast, root mean squared error (RMSE) is sensitive to absolute group differences, and while expressing performance as percent improvement over a null predictor (%ΔRMSE) adjusts for variance, it remains affected by mean shifts between groups. %ΔRMSE is therefore best viewed as a complementary measure, not a replacement for correlation:

$\%\Delta \mathrm{RMSE}=100\times \left(1-\frac{\mathrm{RMS}{\mathrm{E}}_{\mathrm{model}}}{\mathrm{RMS}{\mathrm{E}}_{\mathrm{null}}}\ \right).$

Here, RMSE_null_​ equals the standard deviation of the test target, ie the variability in cognition that a naïve mean predictor cannot reduce. Given the modest effect sizes typical in brain-cognition prediction (Rasero et al. 2021; Schulz et al. 2024), absolute improvements in %ΔRMSE are expected to be small. Note that absolute accuracies are further reduced in our design because splitting the cohort into younger and older halves reduces the training sample size by half, but this is necessary to evaluate age-dependent generalisability. For these reasons, we present correlation as the headline metric in the main text, while RMSE-based metrics are provided in the **Supplementary Information** for completeness.

## Results

We assessed age-dependent differences in the relationship between brain structure and cognition by (i) testing associations between individual IDPs and cognition across age quartiles, and (ii) evaluating the generalisability of multivariate predictive models between age groups.

### Age moderates brain-cognition associations

**Figure 2:**
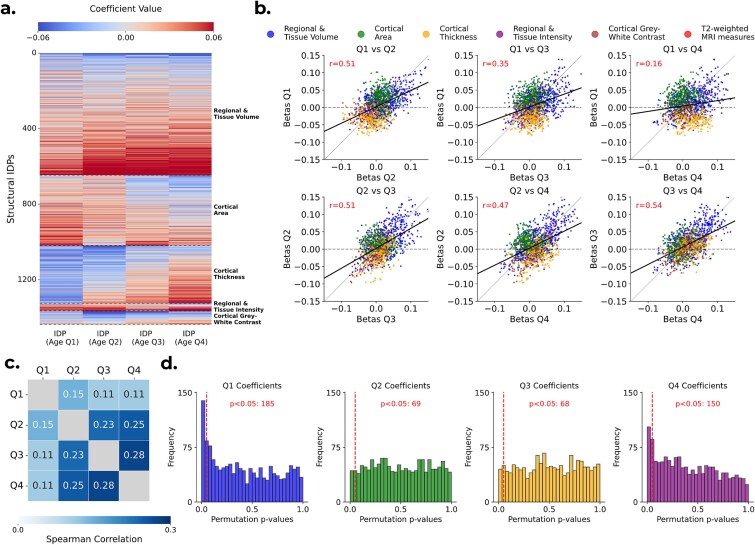
a) Heatmap of coefficients from the linear regression models predicting cognitive performance using each of the 1439 IDPs individually. Each model estimated four quartile-specific IDP coefficients (x-axis), shown across all 1439 IDPs (y-axis), after fully deconfounding all age-related effects from both the IDPs and the cognitive outcome. Structural IDPs are grouped by category (regional and tissue volume, cortical area, cortical thickness, regional and tissue intensity, and cortical Gray-white contrast). Three T2-weighted MRI measures are included at the bottom of the heatmap but are not labeled separately due to their small number. b) Scatterplots comparing quartile-specific IDP coefficients across age quartiles. Each scatterplot compares coefficients between two quartiles: (i) Q1 vs. Q2. (ii) Q1 vs. Q3. (iii) Q1 vs. Q4. (iv) Q2 vs. Q3. (v) Q2 vs. Q4. (vi) Q3 vs. Q4. c) Heatmap of Spearman’s correlations between the absolute values of all IDP coefficients across age quartiles. Darker shades indicate stronger positive correlations, highlighting the particularly low correlation between the youngest quartile (Q1) and the others. d) Histograms of uncorrected *P*-values from permutation testing of the quartile-specific IDP-cognition regression coefficients, with one histogram per age quartile (each based on 1439 IDPs, ie one *P*-value per IDP in that quartile). The red dashed lines mark the *P* = 0.05 threshold. Under the null hypothesis of no coefficient differences across age quartiles, each distribution would be uniform, and approximately 72 *P*-values (5% of 1439) would fall below this threshold by chance.

Using the quartile-stratified regression framework, we compared IDP-cognition associations across age groups. This analysis revealed clear age-dependent patterns in the regression coefficients linking brain structure to cognition. As shown in Fig. 2a, certain IDPs exhibited distinct age-related patterns: for example, cortical thickness IDPs tend to show stronger negative coefficients in the youngest quartile (indicated in blue) and stronger positive coefficients in the oldest quartile (indicated by red). Figure 2b presents scatterplots comparing coefficients between quartiles, highlighting the particularly low Pearson correlation between the youngest (Q1) and oldest (Q4) quartiles (*r* = 0.16). The variance of the cognitive outcome was similar across quartiles (SD ≈ 2.1–2.3; Table S4, Fig. S7), and IDPs were standardized within each quartile, confirming that coefficient differences are not driven by unequal scaling.

As a control analysis, we repeated the quartile-stratified regression without deconfounding for age (Fig. S8). In the absence of age deconfounding, regression coefficients were dominated by shared age-related variance, yielding coefficient patterns that were more consistent in sign and overall structure across quartiles than in the deconfounded analysis (Fig. 2a). Without age deconfounding, structural IDPs can act as proxies for chronological age, such that apparent associations primarily reflect shared age dependence rather than differences in residual brain-cognition relationship. This control analysis supports using explicit age deconfounding to isolate age-dependent differences in residual brain-cognition associations.

These patterns are further summarized in Fig. S9. Fig. S9a summarizes the full coefficient distributions across quartiles, illustrating both the systematic shift in medians and the increased variability in Q4. Figure S9b shows Bland–Altman plots of Fisher-transformed IDP-cognition correlations across quartile pairs, revealing larger between-quartile divergence for comparisons involving Q1 and Q4.

We next asked whether the relative importance of different brain features changed across age groups. To investigate this, we used the absolute value of the coefficients as a proxy for feature importance and computed Spearman’s correlation between quartiles. Figure 2c shows the resulting heatmap, indicating that the most predictive IDPs in Q1 (ie the youngest subjects) differ from those in older subjects. Specifically, the correlations between Q1 and the other quartiles were particularly low (Q1 vs. Q2: r_s_ = 0.15; Q1 vs. Q3: r_s_ = 0.11; Q1 vs. Q4: r_s_ = 0.11), further emphasizing the weak resemblance between Q1 and the older age groups. The corresponding Pearson analysis is shown in Fig. S9c, showing the same pattern.

Permutation testing of the IDP-cognition regression coefficients (see **Methods**) produced 5756 *P*-values (1439 IDPs × 4 quartiles). After Benjamini–Hochberg FDR correction across all IDP-quartile tests, no individual IDP exhibited a statistically significant difference in association across age quartiles. This pattern is consistent with the presence of weak and distributed associations rather than a small number of dominant features. Nevertheless, the largest age-moderation effects were concentrated in comparisons between the youngest and oldest quartiles. Figure S10 summarizes IDP-level effect sizes for ${\beta}_{Q4}-{\beta}_{Q1}$ against permutation evidence (Fig. S10a), suggesting that these effects are most consistently expressed in cortical thickness measures at the category level (Fig. S10b).

We therefore next assessed whether age-dependent differences across quartiles were present at the population level, rather than at the level of individual IDPs, by testing for systematic deviations in the distribution of permutation *P*-values.

Figure 2d shows the distributions of uncorrected permutation *P*-values across IDPs for each age quartile. These deviated from uniformity at the distributional level for the youngest and oldest quartiles (Q1: *p*_KS_ < 0.001; Q4: *p*_KS_ < 0.001), but not for the middle quartiles (Q2: *p*_KS_ = 0.66; Q3: *p*_KS_ = 0.09). This indicates that age-dependent differences are concentrated at the extremes of the age distribution.

To quantify this effect at the distributional level, we applied two complementary global enrichment analyses (Methods S3). First, Storey’s ${\pi}_0$ estimates indicated a substantial excess of non-null effects in Q1 (${\pi}_0$= 0.81) and Q4 (${\pi}_0$ = 0.62), but not in Q2 (${\pi}_0$ = 0.97) or Q3 (${\pi}_0$= 0.99). Second, a permutation-based Fisher’s combined test confirmed strong global enrichment in Q1 and Q4 (both *P* < 1 × 10^−4^), with no evidence of enrichment in Q2 or Q3. Together, these results indicate that age-dependent differences across quartiles are concentrated at the extremes of the age distribution and are detectable at the level of the IDP ensemble, even though individual IDPs do not survive multiple-comparison correction.

In summary, beyond general age effects, the overall pattern of structural IDP–cognition associations differed across age groups, with evidence concentrated at the extremes of the age distribution and apparent at the distributional level despite no single IDP surviving multiple-comparison correction. This supports our hypothesis that between-subject variations in brain structure associated with variations in cognition in older adults may be different from those in younger adults.

### Age-specific models outperform pooled and cross-age generalization

**Figure 3:**
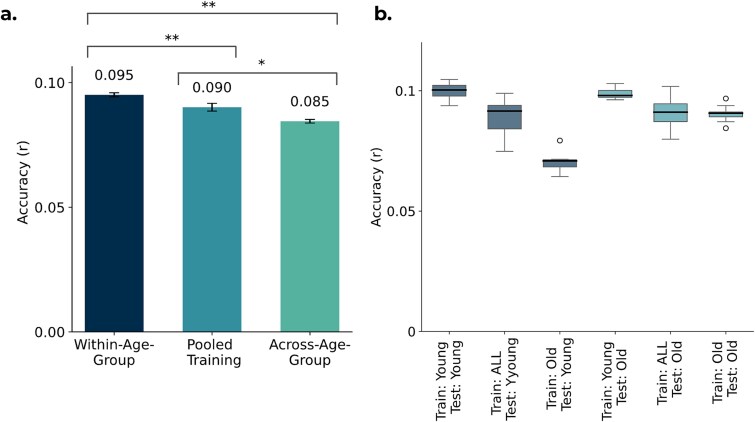
Comparison of prediction performance across different training strategies when using 1439 structural IDPs to predict cognition. Models were evaluated using 10-fold cross-validation repeated 10 times, yielding 10 matched out-of-sample performance estimates shared across all training/testing regimes. Both the IDPs and cognitive trait were deconfounded for all confounds (including age-related terms). Prediction accuracy is measured as the Pearson correlation (r) between predicted and observed cognitive scores. a) Mean prediction accuracy for three training/testing regimes: within-age-group (Train: Young, Test: Young; and Train: Old, Test: Old), across-age-group (Train: Young, Test: Old and Train: Old, Test: Young), and pooled training (Train: ALL, Test: Young; and Train: ALL, Test: Old). For each repetition, accuracy was averaged across the relevant train-test splits using Fisher’s z transformation. Bars show the mean across repetitions; error bars indicate the standard error of the mean. Statistical annotations indicate two-sided paired Wilcoxon signed-rank tests across repetitions with Benjamini-Hochberg FDR correction across planned contrasts (^*^P < 0.05, ^**^P < 0.01). b) Comparison of prediction accuracy across six training/testing splits, with models trained and tested on different age groups. Boxplots show the distribution across cross-validation repetitions.

Using the multivariate elastic net framework, we systematically compared the out-of-sample prediction accuracy of the three training strategies: within-age-group, across-age-group, and pooled models. This analysis revealed a clear and consistent performance hierarchy, as shown in Fig. 3a. Age-specific models achieved the highest accuracy (r_WITHIN-AGE-GROUP_ = 0.095), significantly outperforming both pooled models (r_POOLED_ = 0.090; *P* = 0.003) and, most strongly, models trained on a different age group (r_ACROSS-AGE-GROUP_ = 0.085; *P* = 0.003). Although these effect sizes are modest, they were consistent and statistically reliable. Pooled models, trained on a mix of ages, offered a robust intermediate performance, in turn proving more accurate than the across-age-group models (*P* = 0.04). This consistent ordering (**within-age-group > pooled > across-age-group**) highlights the clear advantage of training models on data that is most representative of the test population.

Consistent patterns were observed when accuracy was expressed as improvement over a null RMSE (%ΔRMSE; Fig. S11b; raw RMSE shown for completeness in Fig. S11a). Within-age-group models achieved the highest gains, followed closely by pooled models, while across-age-group models performed substantially worse. Absolute improvements were small (<1%) as expected given the modest effect sizes typical in this domain.

### Younger-trained models generalize across age groups

We next examined whether the observed effects were symmetrical across age groups. Figure 3b shows that models trained on younger subjects consistently outperformed those trained on older subjects, regardless of the testing group. Although the gap was modest when predicting older subjects (r_TR: Y; TE: O_ = 0.099 vs. r_TR: O; TE: O_ = 0.090; *P* = 0.002; mean Δr = 0.008, 95% CI [0.007, 0.010]), the gap was more pronounced for younger subjects, where older-trained models were notably less accurate (r_TR: Y; TE: Y_ = 0.100 vs. r_TR: O; TE: Y_ = 0.070; *P* = 0.002; mean Δr = 0.029, 95% CI [0.026, 0.033]; Fig. S12). This suggests that models trained on older subjects struggle to capture brain-cognition relationships relevant to younger individuals, whereas those trained on younger subjects remain predictive even for older individuals.

Figure 3b further shows that the effect of training strategy depended on the test group. For younger subjects, within-age-group models achieved the highest accuracy, outperforming both pooled (r_TR: Y; TE: Y_ = 0.100 vs. r_TR: ALL; TE: Y_ = 0.089) and across-age-group models (r_TR: Y; TE: Y_ = 0.100 vs. r_TR: O; TE: Y_ = 0.070; *P* = 0.002). For older subjects, performance was more similar across conditions: within-age-group and pooled models performed comparably (r_TR: O; TE: O_ = 0.090 vs. r_TR: ALL; TE: O_ = 0.091; *P* = 1.00), while younger-trained models outperformed pooled models (r_TR: Y; TE: O_ = 0.099 vs. r_TR: ALL; TE: O_ = 0.091). Importantly, the correlation-based pattern was robust to alternative, more conservative cognitive composite construction strategies, including independent trait selection and a composite built from all traits with high coverage (Fig. S6).

RMSE-based accuracy metrics are provided in **Fig. S11c** and **S11d**. These broadly supported the correlation-based findings, while also illustrating how between-group variance differences can affect RMSE-based measures.

Overall, these results demonstrate a key asymmetry in generalization, where brain-cognition patterns from a younger population serve as a more generalisable model, while patterns unique to an older population do not. These analyses highlight a trade-off between model generalisability and age-group specificity.

### Older-trained models favor stronger regularization

**Figure 4:**
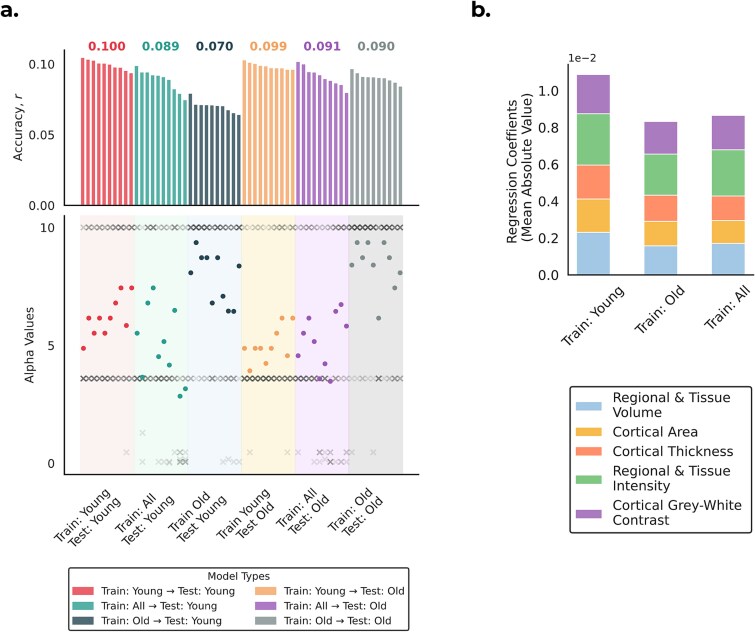
Overview of model regularization and feature importance across training strategies. a) Comparison of elastic net regularization strength and model accuracy. The top panel shows mean prediction accuracy across cross-validation repetitions (numbers above bars indicate the mean r). The bottom panel shows the regularization strength (α) per cross-validation repetition (× = individual folds; ● = mean across 10 folds). Hyperparameters were determined via nested cross-validation, with the inner loop selecting the optimal α. b) Stacked bar plot showing the mean absolute values of regression coefficients, averaged across IDPs within each structural category, for the three training approaches.

To better understand these performance differences, we examined the models’ hyperparameters. As shown in Fig. 4a, models trained on older subjects consistently selected higher levels of regularization (α) than those trained on the full population, both when tested on younger subjects ($\alpha$_TR: O; TE: Y_ = 7.87 vs. $\alpha$_TR: ALL; TE: Y_ = 4.97) and on older subjects ($\alpha$_TR: O; TE: O_ = 8.40 vs. $\alpha$_TR: ALL; TE: O_ = 5.17). By contrast, models trained on younger subjects used lower levels of regularization, closer to pooled models ($\alpha$_TR: Y; TE: O_ = 5.00; $\alpha$_TR: Y; TE: Y_ = 6.20).

These results suggest that models trained on older subjects tend to select stronger regularization to avoid overfitting, consistent with higher noise levels or greater inter-subject variability in this group. Models trained on younger subjects, or on pooled samples that include younger individuals, require less regularization, which may explain why pooled models perform comparably to those trained only on older subjects. Although including younger subjects in pooled models may introduce distributional bias when testing on older subjects, their lower noise levels reduce the need for regularization and help preserve predictive signal.

An analogous analysis of the elastic-net mixing parameter (ρ; equivalent to the L1 ratio in scikit-learn) is provided in Fig. S13. Age-specific models (Train: Young and Train: Old) consistently selected ridge-like solutions (L1 ratio ≈ 0, ie favoring shrinkage of all coefficients rather than sparsity), whereas pooled models showed small but non-zero L1 ratios (0 to 0.25). This indicates that the principal difference across training strategies lies in the strength of regularization (α), rather than in the balance between L1 and L2 penalties.

Figure 4b shows that models trained on older subjects had consistently smaller mean absolute regression coefficients across IDP categories, relative to younger-trained models. The largest relative reductions were observed in Regional and Tissue Volume (31.7% across 646 IDPs), Cortical Area (26.5% across 372 IDPs), and Cortical Thickness (22.9% across 306 IDPs). Smaller but consistent reductions appeared in Regional and Tissue Intensity (19.8% across 41 IDPs) and Cortical Gray-White Contrast (16.9% across 70 IDPs). Given the larger number of IDPs in the first three categories, these reductions are likely to have the greatest impact on model performance. Pooled training produced intermediate values across all categories, consistent with the idea that including a broader age range stabilizes coefficient estimates by averaging over age-specific variability.

## Discussion

Accounting for the influence of age is essential when studying brain-cognition relationships. While the standard approach is to remove age as a confound, we suggest it is more appropriate to treat age as a moderator, finding that the statistical links between brain structure and cognition vary significantly across the lifespan. Importantly, we use “moderation” broadly to refer to age-dependent reorganization of brain-cognition relationships, which is not necessarily well captured by the orthodox approach of fitting a single linear age × IDP interaction term.

Although absolute prediction accuracies were modest (r ≈ 0.07–0.10), they are consistent with prior large-sample neuroimaging studies after accounting for sample size and deconfounding (Rasero et al. 2021; Schulz et al. 2024). Our focus was not maximizing absolute accuracy, but assessing whether the brain-cognition relationships generalize across age groups. This question revealed clear age-dependent differences in generalisability.

A key design choice was to treat age as a confound and regress out direct age effects to avoid results being driven by shared age dependence in IDPs and cognition. We then test whether the residual brain-cognition mapping differs across age groups using stratified and cross-age generalization analyses. In control analyses without age deconfounding, associations largely track chronological age (ie IDPs act as proxies for age), making age-group differences harder to detect.

### Age-dependent reorganization of brain-cognition associations

The quartile-based univariate analyses indicate that brain-cognition relationships differ across the adult lifespan. Coefficients and feature-importance rankings showed only modest agreement across quartiles, with the weakest consistency at the extremes of the age distribution. The overall signal was weak and distributed rather than driven by a small set of dominant IDPs, and sign reversals in some feature classes (eg cortical thickness) suggest that the structural correlates of cognition shift with age, potentially reflecting greater heterogeneity in aging-related processes later in life. Together, these findings provide a univariate perspective on why cross-age prediction may not transfer uniformly between age groups.

### Generalization asymmetry and its link to age-related variability

Previous studies have reported age-related differences in prediction generalisability (Yu and Fischer 2022); we extend this in UKB using an age-stratified generalization framework. We observed an asymmetry in how predictive models generalize across age. Models trained on younger subjects generalized well to older subjects, likely capturing foundational brain-cognition relationships that remain stable with age. In contrast, models trained on older subjects generalized poorly to younger individuals.

Our analysis of the model’s hyperparameters suggests one possible reason for this asymmetry. Older-trained models consistently selected stronger regularization to prevent overfitting, which is consistent with higher noise levels (Nomi et al. 2024) or greater inter-subject variability in this group. Including younger subjects, as in the pooled models, appears to stabilize coefficients by reducing this need for high regularization. This suggests that the brain-cognition relationship in older age is complicated by additional sources of variance—such as differential neurodegeneration or comorbidities—making it harder to learn a pattern that generalizes back to the more homogeneous younger population. Consistent with this, within-age-group models performed better than across-age-group models. This highlights the benefit of training on data matched to the test set distribution.

Practically, this suggests a trade-off between specialization and robustness: age-specific models can maximize within-group accuracy but may be unstable out-of-distribution, especially when trained on older participants. By contrast, pooled training provides more consistent generalization across age groups, and may be preferable for heterogeneous cohorts or when the target age range is uncertain.

### Limitations and next steps

We concentrated on structural IDPs for their robust age trends, high test–retest reliability, larger UKB sample coverage than fMRI, and reduced sensitivity to age-related motion. However, functional connectivity can yield higher prediction accuracy (Ooi et al. 2022; Soch et al. 2022).

In this work, we focused on the first principal component derived from cognitive traits. However, cognition comprises multiple subdomains, and age may influence structure–cognition associations differently across domains (Van Petten 2004; Yuan and Raz 2014; Cacciaglia et al. 2018). An important next step is to repeat these analyses using domain-specific composites to test the generality of the age-stratified patterns.

Poorer generalization in older-trained models may partly reflect the higher prevalence of age-related comorbidities (eg hypertension, diabetes, cardiac disease), which can affect brain structure (Cox et al. 2019). Robustness could be assessed by restricting analyses to healthier participants and/or adjusting for these conditions. In addition, future work could account for global or regional atrophy (eg total gray matter volume or regional volume measures) and explicitly quantify how much of the age-dependent association is mediated by atrophy versus residual variance beyond atrophy, helping distinguish decline-related from non-decline-related effects.

Finally, UKB spans mid-life to older adulthood, so we cannot test whether the same age-stratified patterns emerge in younger adults or other typically developing cohorts. With the increasing availability of datasets that include younger age ranges (eg the Human Connectome Project—Young Adult (HCP-YA)), future studies could apply the same framework to earlier stages of the lifespan to test whether similar cross-age generalization patterns are observed. Alongside broader age coverage, continuous age-varying coefficient models could capture nonlinear effects more precisely and help identify specific ages at which brain-cognition relationships change, and could be combined with stronger confound control (eg propensity score matching; Rosenbaum and Rubin 1983) to improve robustness (Power et al. 2012).

## Conclusion

This work demonstrates that the link between brain structure and cognition is not fixed but changes across the adult lifespan. A central finding was an asymmetry in generalisability: models trained on younger subjects predicted cognition in older individuals, whereas models trained on older subjects did not generalize to younger individuals. This reveals an important trade-off for researchers and clinicians building predictive models, and the optimal approach depends directly on the intended goal.

For example, in situations requiring high accuracy within a specific target group (eg clinical diagnostics), an age-specific model may be preferable. However, in situations requiring a model to generalize reliably across a diverse, mixed-age population, a pooled model trained on all ages provides more stable and broadly applicable predictions. Recognizing this trade-off is essential for improving prediction models in both research and clinical settings, ensuring that predictions are optimal for the intended population.

## Supplementary Material

CerCor_-_2025_-_00722_Supplementary_Information_R1_bhag024_S1_UPDATED_docx

## Data Availability

Data were obtained from the UK Biobank under application 8107. Access to UK Biobank data is available to bona fide researchers via application to UK Biobank (https://www.ukbiobank.ac.uk/enable-your-research). Derived data supporting the findings of this study are available from the corresponding author upon reasonable request, subject to UK Biobank’s data access policies.
