# Supplementary material for: The role of age in the relationship between brain structure and cognition: moderator or confound?
Source: Cereb Cortex. 2026 Mar 11;36(3):bhag024. doi: 10.1093/cercor/bhag024 (PMC13017657; doi:10.1093/cercor/bhag024)
Supplement: CerCor_-_2025_-_00722_Supplementary_Information_R1_bhag024_S1_UPDATED_docx [file cercor_-_2025_-_00722_supplementary_information_r1_bhag024_s1_updated_docx.docx]

Supplementary Data

The role of age in the relationship between brain structure and cognition: moderator or confound?

Ben Griffin^1,2,3*^, Chetan Gohil^3^, Mark W. Woolrich^3^, Stephen M. Smith^1^ and Diego Vidaurre^2,3,4^

^1^Oxford Centre for Functional MRI of the Brain (FMRIB), Oxford Centre for Integrative Neuroimaging, Nuffield Department of Clinical Neurosciences, University of Oxford, John Radcliffe Hospital, Headley Way, Headington, Oxford, Oxfordshire OX3 9DU, United Kingdom

^2^Center of Functionally Integrative Neuroscience, Department of Clinical Medicine, Aarhus University, Building 1710, Universitetsbyen 3, 8000 Aarhus, Denmark
^3^Oxford Centre for Human Brain Activity (OHBA), Oxford Centre for Integrative Neuroimaging, Department of Psychiatry, University of Oxford, Warneford Hospital, Warneford Lane, Headington, Oxford, Oxfordshire OX3 7JX, United Kingdom

^4^Centre de Recerca Matemàtica, Campus UAB, Carrer de l'Albareda, Edifici C, 08193 Bellaterra, Barcelona, Spain

***Corresponding author**

**Correspondence:**

Ben Griffin

Oxford Centre for Functional MRI of the Brain (FMRIB)

Oxford Centre for Integrative Neuroimaging

Nuffield Department of Clinical Neurosciences, University of Oxford

John Radcliffe Hospital

Headley Way, Headington

Oxford, Oxfordshie OX3 9DU

United Kingdom

Telephone: +44 (0)1865 234829

Email: ben.griffin@keble.ox.ac.uk

**Supplementary Information**

**Supplementary Methods**

**Supplementary Methods S1: Construction of the composite cognitive measure**

### **S1.1 Trait screening and selection**

In brief, we (i) filtered traits by coverage, (ii) ranked remaining traits by cross-validated brain-cognition predictability, (iii) selected the top 30 based on an inflection in the ranked curve, and (iv) used the first principal component (PC1) from these traits as the composite target (**Supplementary Figure S5**).

We constructed a composite cognitive target to improve predictive stability and statistical power relative to individual cognitive measures. We excluded cognitive traits with >50% missing responses (i.e., <50% coverage). For each remaining trait, we assessed predictability by fitting elastic net regression models using all 1,439 structural IDPs as predictors and evaluating out-of-sample performance via cross-validation. Traits were then ranked by their cross-validated brain–cognition correlation.

Inspection of the ranked correlation curve revealed a clear inflection around rank ≈30, beyond which predictive performance dropped sharply (**Supplementary Figure S5a,b**). We therefore selected the top 30 traits as candidate targets. This threshold balances retaining traits with meaningful brain-cognition signal while avoiding the inclusion of poorly predicted measures that would add noise and reduce power. The resulting trait set spans a broad range of UK Biobank cognitive measures (**Supplementary Table S5**). Pairwise correlations among these traits indicate limited redundancy, with many trait pairs only weakly correlated (**Supplementary Figure S5c**), suggesting that the set captures multiple cognitive dimensions rather than a single narrow construct.

### **S1.2 Composite construction**

To summarise shared variance across the selected traits and further stabilise prediction, we derived a composite cognitive score by applying principal component analysis (PCA) to the 30 selected traits. All traits were standardised prior to PCA. The first principal component was retained as the composite cognitive measure, as it captured the largest proportion of shared variance and achieved higher predictive accuracy than any individual trait (**Supplementary Figure S5d**). This composite therefore provides a more robust and informative target for prediction analyses than single cognitive measures.

While conceptually related to general intelligence (g-factor), this composite differs in that it is derived only from traits that are demonstrably predictable from brain structure, rather than from the full set of available cognitive measures.

### **S1.3 Cross-validation and leakage considerations**

Trait selection was performed once, outside the main cross-validated modelling framework, using all available subjects. This choice was made to ensure consistency of the cognitive target across models and to avoid the substantial computational burden and interpretational complexity that would arise from re-selecting traits within each cross-validation fold. This can modestly inflate absolute prediction accuracy (which we address via the sensitivity analyses in **Supplementary Figure S6**) but is unlikely to materially affect comparisons across training/testing strategies because the same fixed target is used for all models. Importantly, trait selection did not involve age stratification or age-dependent modelling and therefore did not condition on age group.

All subsequent modelling steps were performed within cross-validation folds to prevent information leakage. Specifically, deconfounding parameters and PCA loadings were estimated on training data and applied to held-out data, and predictive models were fit on training data and evaluated on held-out subjects. As a result, while the identity of the selected traits was fixed, the composite scores (PC1) and all predictive evaluations were computed strictly out-of-sample.

**Supplementary Methods S2: Permutation testing of age-moderated effects**

To test whether IDP-cognition associations differed across age quartiles, we used a permutation-based inference procedure applied to the quartile-stratified univariate regression coefficients.

For each IDP $i$, the quartile-based regression yielded four IDP coefficients $\beta_{i,1},\ldots,\beta_{i,4}$, corresponding to age quartiles Q1-Q4. For each quartile $k$, we defined a contrast statistic

$$D_{i,k}=\mid\beta_{i,k}-\frac{1}{3}\sum_{k^{'}\neq k} \beta_{i,k^{'}}\mid,$$

which quantifies the deviation of the quartile-specific association from the average association in the remaining three quartiles. This statistic is symmetric across quartiles and equals zero under the null hypothesis of identical coefficients across age groups.

Null distributions for $D_{i,k}$ were generated by randomly permuting subjects’ age-quartile assignments (i.e., permuting quartile labels across subjects, equivalently shuffling group membership, which preserves the sample size in each quartile) while keeping all other aspects of the model fixed. This procedure was repeated 10,000 times per IDP, yielding a permutation distribution for each quartile-specific contrast.

Permutation p-values were computed as

$$p_{i,k}=\frac{1+\sum_{n=1}^{N} \mathbb{I}\left( D_{i,k}^{\left( n \right)} \geq D_{i,k} \right)}{N+1},$$

where $N=10,000$, and the +1 correction prevents zero p-values.

Under the null hypothesis of no age-dependent differences in brain-cognition associations, these p-values are expected to follow a uniform distribution. Deviations from uniformity across IDPs therefore indicate systematic age moderation at the population level.

**Supplementary Methods S3: Global enrichment analyses**

Because stringent multiple-comparison correction can limit sensitivity when testing large numbers of features, we additionally assessed evidence for age-moderated effects at the population level using three complementary global enrichment analyses applied to the permutation-derived p-values from the quartile-based permutation tests described in **Supplementary Methods S2**.

***S3.1 Kolmogorov-Smirnov test for p-value uniformity***

To assess whether permutation-derived p-values deviated from the Uniform(0,1) distribution expected under the global null hypothesis, we applied a Kolmogorov-Smirnov (KS) test separately within each age quartile. The KS test compares the empirical cumulative distribution function of observed p-values to the theoretical uniform distribution and provides a sensitive diagnostic of population-level deviation from the global null. Significant deviations from uniformity indicate systematic deviation from the global null across IDPs, consistent with age-dependent effects across IDPs.

#### **S3.2 Storey’s π₀ estimation**

To estimate the proportion of IDPs exhibiting non-null age-moderated effects, we applied Storey’s method to the permutation p-values within each age quartile (Storey, 2002). This approach models the observed p-value distribution as a mixture of a uniform null component and a non-null component, yielding an estimate of $\pi_{0}$, the proportion of true null hypotheses. Lower values of $\pi_{0}$ indicate greater enrichment of non-null effects. For a tuning parameter λ, we estimated

$$\hat{\pi}_{0}\left( \lambda\right)=\frac{\#\left\{ p_{i}>\lambda\right\}}{m(1-\lambda)}.$$

Here $p_{i}$ denotes the permutation p-value for IDP $i$ and $m$ is the number of IDPs. We evaluated a high-$\lambda$ grid $\lambda\in[0.5, 0.9]$ and retained a stable estimate (Storey & Tibshirani, 2003).

#### **S3.3 Permutation-based Fisher global test**

As a complementary analysis, we assessed global enrichment using Fisher’s method to combine p-values across IDPs within each age quartile (Fisher, 1970). This analysis tests the global null hypothesis that all IDP-quartile associations are null, and does not aim to identify individual non-null IDPs. For each quartile, the Fisher statistic was computed as

$$S=-2\sum_{i=1}^{m} \log(p_{i}),$$

where $p_{i}$ denotes the permutation p-value for IDP $i$, and $m=1,439$.

To account for dependence among IDPs, statistical significance was assessed using permutation-based inference rather than the theoretical chi-squared distribution(Nichols & Holmes, 2002). Specifically, for each permutation we recomputed the Fisher statistic using the set of p-values obtained under that permutation, yielding an empirical null distribution for $S$. The global p-value was defined as the proportion of permutations in which the permuted Fisher statistic exceeded the observed value, using a +1 correction to avoid zero p-values.

Together, these global enrichment analyses provide complementary population-level evidence for age-moderated brain-cognition associations, even in the absence of individually significant IDPs after multiple-comparison correction.

**Supplementary Figures**

**Supplementary Figure S1. Audit of missing structural IDPs**

The number of missing structural IDPs per subject showed only weak associations with age (Pearson r = 0.034) and cognition (Pearson r = 0.025). When stratified by age quartiles, median missingness was stable (Q1-Q3: median 2 missing IDPs; Q4: median 3). Although the proportion of participants with any missing IDP was modestly higher in the oldest quartile (84.5% vs 76.5-79.6%), this reflects small increases in low-level missingness rather than substantial differences in missing data burden. Given the large sample size, these effects are statistically detectable but small in magnitude.***
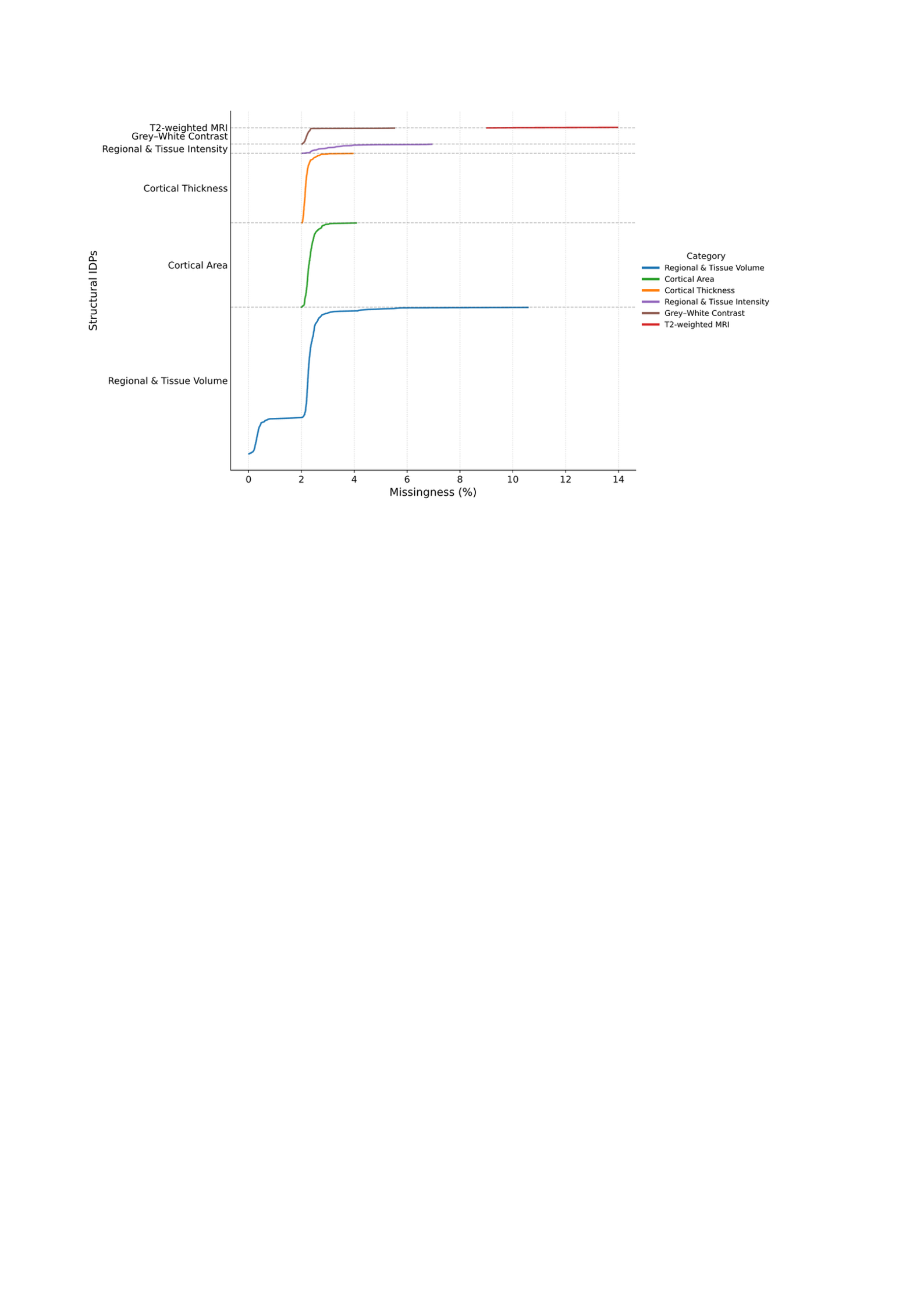
***

***Figure S1.*** *Distribution of missing values across structural imaging-derived phenotypes (IDPs)*. Shown is the percentage of missing values per IDP, grouped by structural category and ordered within each category by increasing missingness. Each point along the curves corresponds to a single IDP. Missingness is low overall and distributed across structural categories, with no single category dominating missing data.

**Supplementary Figure S2.** Age distribution and quartile boundaries used for age-stratified analyses

**
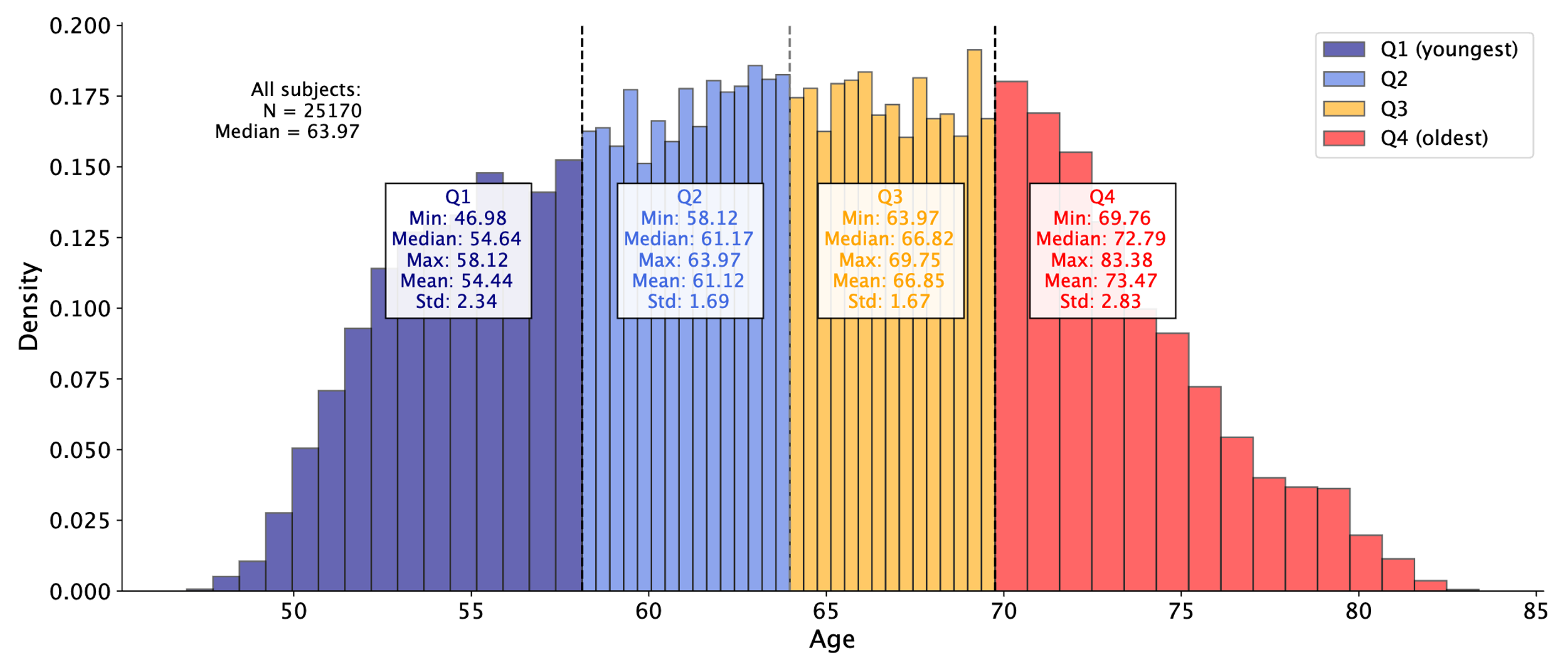
**

***Figure S2.*** *Histogram of participant age distributions, divided into quartiles.* Each colour-coded section corresponds to one of the four age quartiles used in the analysis (Q1: youngest, Q4: oldest). Vertical dashed lines indicate the boundaries between quartiles.

**Supplementary Figure S3.** Age distributions by sex within younger and older halves of the UK Biobank cohort

**
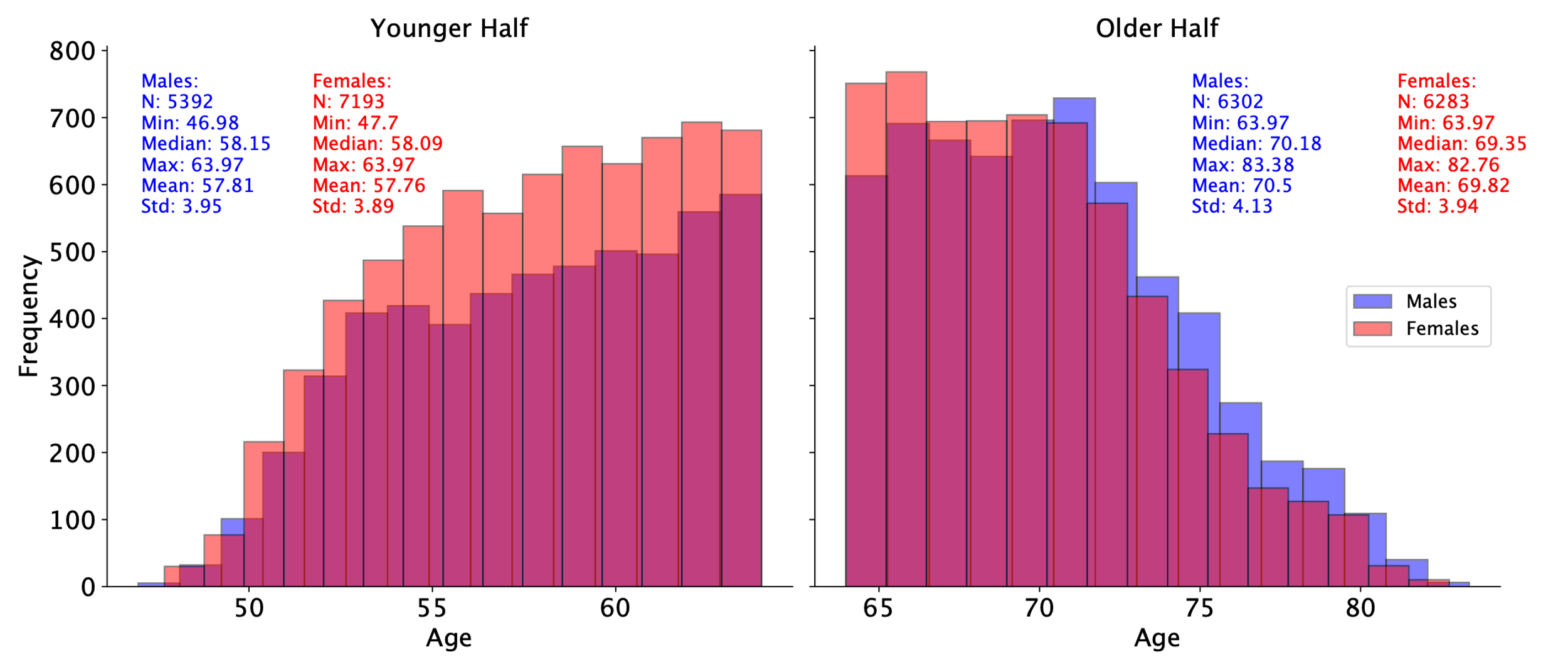
**

**Figure S3.** Histogram of age distributions in UKB subjects, split by sex within each age group. Both younger and older halves contain approximately equal numbers of males and females, with similar age distributions across sexes. This confirms that observed effects are unlikely to be driven by imbalanced age-sex sampling.

**Supplementary Figure S4.** Distribution of incremental age-related variance across structural IDPs

**
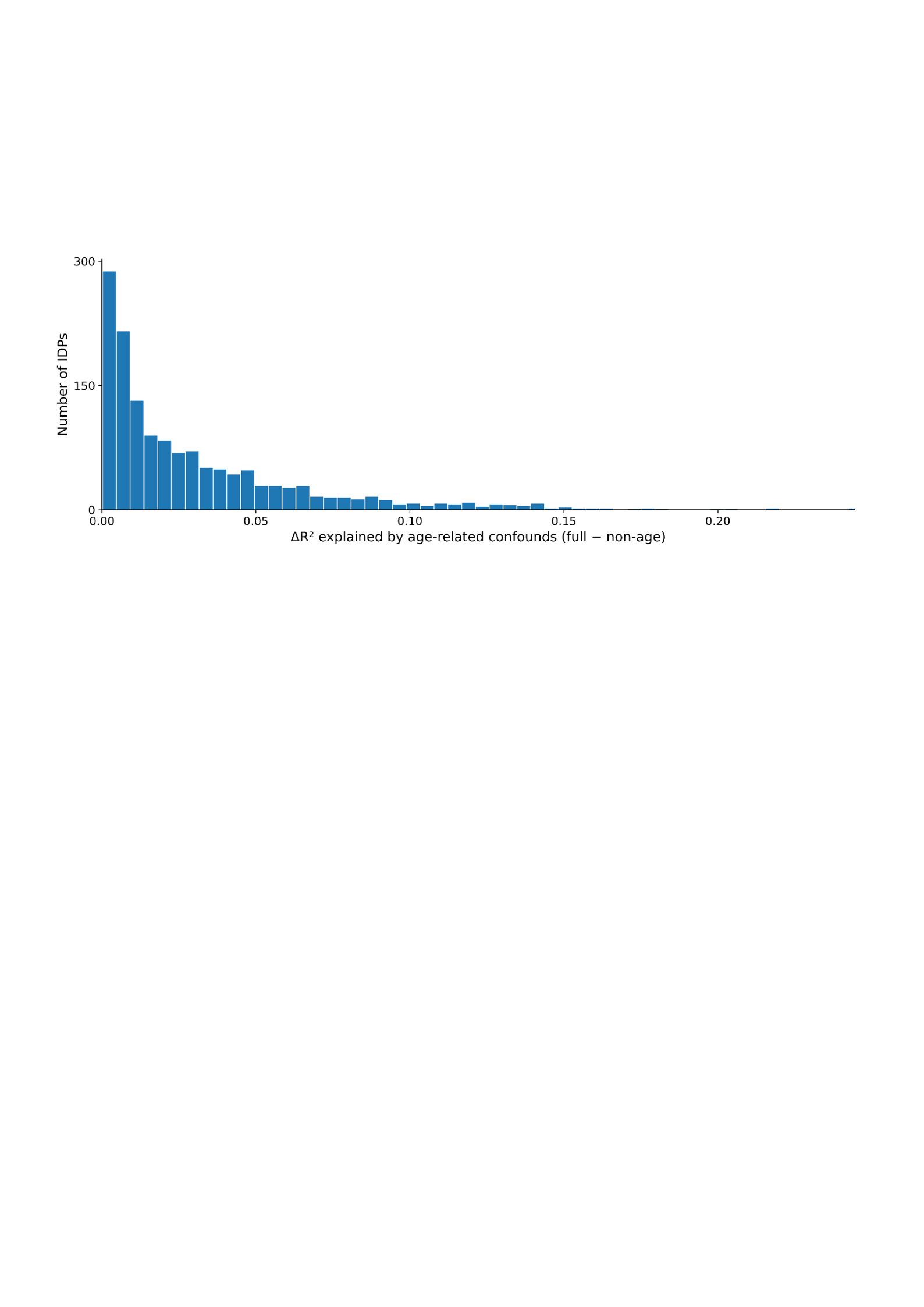
**

**Figure S4.** Incremental variance explained by age-related confounds across structural IDPs. For each IDP, we computed the increase in variance explained when adding age-related terms to the confound model (ΔR² = R²_full − R²_non-age), where the “full” model includes age-related confounds and the “non-age” model includes all other confounds only. The distribution is right-skewed: for most IDPs, age-related terms explain only a small additional proportion of variance, with a minority of IDPs showing larger incremental age-related contributions.

**Supplementary Figure S5.** Selection and dimensionality reduction of cognitive traits

**Supplementary Figure S5** summarises the trait-selection and PCA steps used to construct the composite cognitive target (**Supplementary Methods S1**).


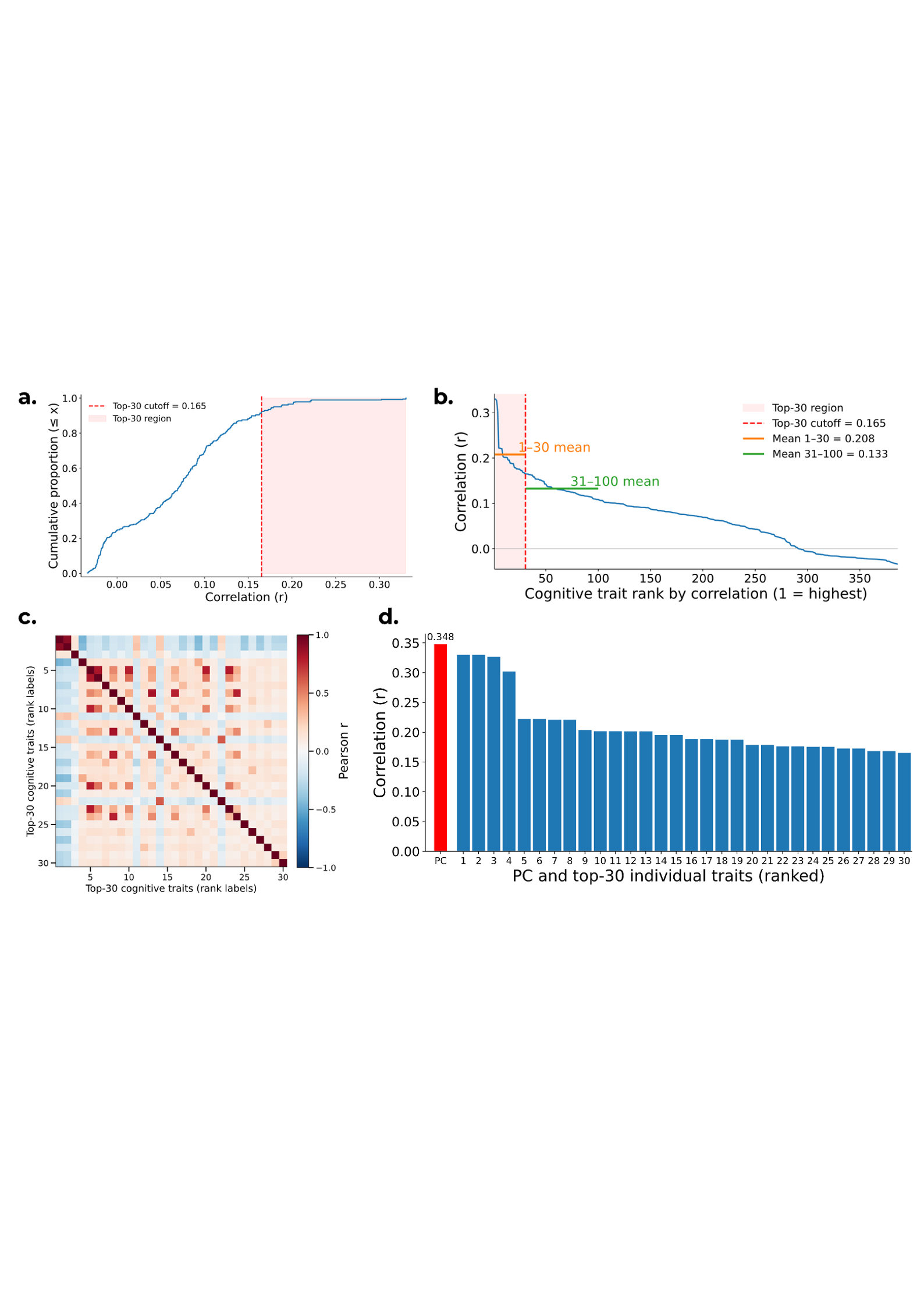


**Figure S5.** Selection and reduction of cognitive traits for prediction analyses. **(a)** Empirical cumulative distribution function (ECDF) of cross-validated brain-cognition correlations for all cognitive traits meeting the coverage criterion (≥50% valid responses per trait); the dashed line marks the 30th-highest correlation. **(b)** Ranked plot of individual trait correlations (rank 1 = highest). The dashed line indicates rank 30; horizontal lines show mean correlations for ranks 1-30 and 31-100. **(c)** Pairwise correlation matrix of the top 30 traits, showing limited redundancy across the set. **(d)** Predictive accuracy (cross-validated r) for the top 30 individual traits (blue) compared with the first principal component (PC1; red) computed from these traits. PC1 achieves higher accuracy and is used as the final cognitive score.

**Supplementary Figure S6.** Sensitivity analyses for cognitive composite construction

In the main analyses, we constructed a composite cognitive target by selecting the 30 cognitive traits most predictable from structural IDPs and then applying PCA to those traits. Because this selection was performed using the full dataset, it may modestly inflate absolute prediction accuracy. To test whether our conclusions depend on this choice, we ran two sensitivity analyses (**Supplementary Figure S6**): (i) selecting traits using an independent 30% subset (age-stratified) and evaluating models on the remaining 70% (**panel a**), and (ii) constructing a composite without trait selection by applying PCA to all cognitive traits with ≥90% coverage (**panel b**). In both cases, the qualitative pattern is preserved: within-age-group models outperform across-age-group models, and models trained on younger participants generalise better to older participants than vice versa.


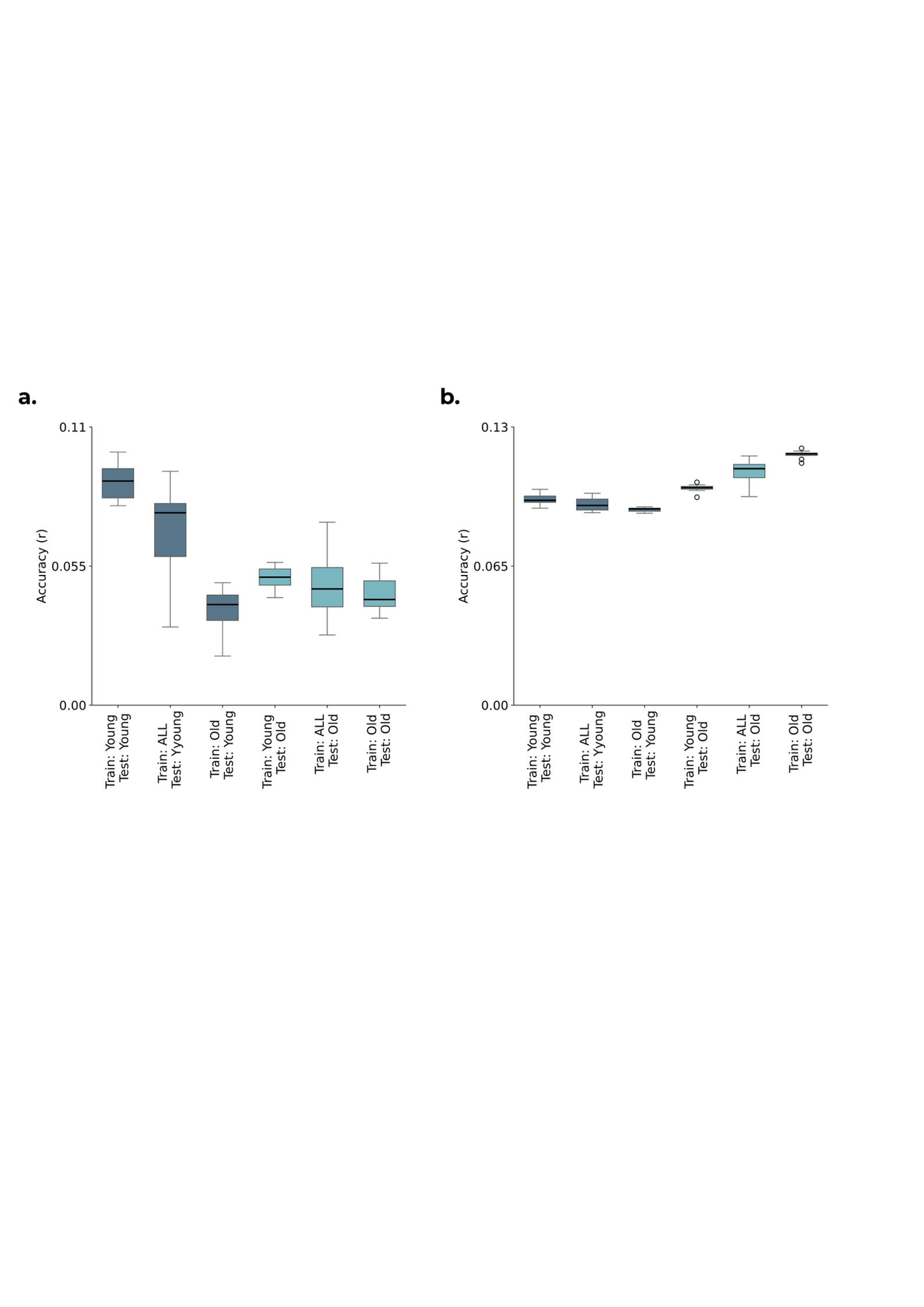


**Figure S6. Sensitivity analyses for cognitive composite construction.** **(a) Trait selection was performed on an independent 30% subset of the cohort (age-stratified), and all models were trained and evaluated on the remaining 70% of participants using the same prediction framework as the main analysis. (b) A non-selective composite target was constructed by retaining all cognitive traits with ≥90% coverage and combining them using PCA without trait selection. Across both analyses, the qualitative ordering of training strategies matches the main results (within-age-group > pooled > across-age-group), and younger-trained models generalise better to older participants than the reverse.**

**Supplementary Figure S7.** Distribution of the composite cognitive score across age quartiles

**Supplementary Figure S7** shows the distribution of the composite cognitive score across age quartiles. The spread of scores is similar across groups (SD≈2.09-2.31; **Table S4**), suggesting that between-quartile coefficient differences are unlikely to be driven by unequal outcome variance.

**
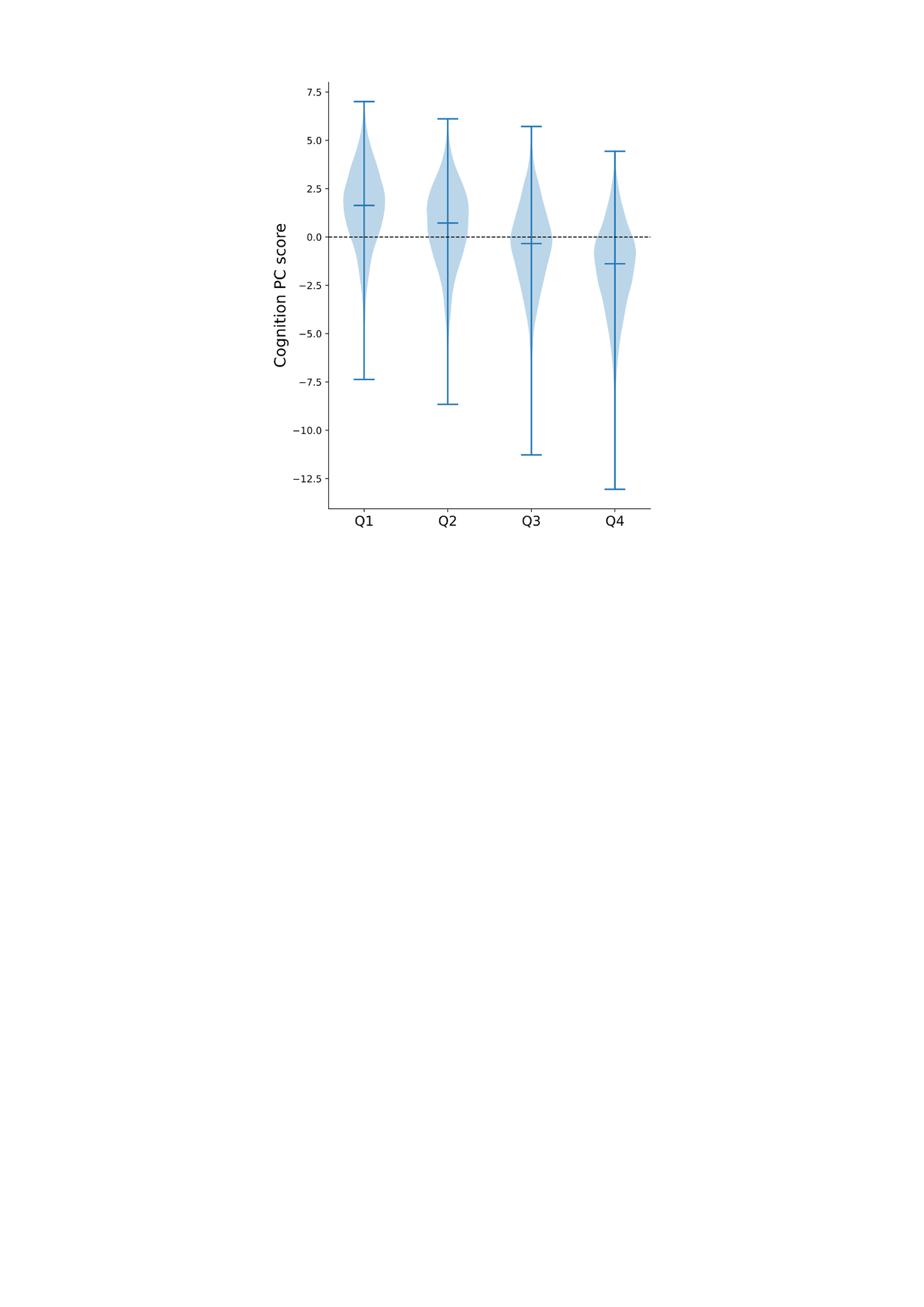
**

**Figure S7.** Distribution of the composite cognitive measure across age quartiles. Violin plots show the composite cognitive score (PC1 of the 30 selected traits) within each age quartile (Q1–Q4). Although the location of the distributions shifts across quartiles, their dispersion is comparable (SD: Q1 = 2.09, Q2 = 2.13, Q3 = 2.24, Q4 = 2.31; Table S4), indicating that quartile-specific coefficient differences are not explained by large differences in target variance. The sign of PC1 is arbitrary, so the direction of the mean shift reflects the chosen orientation of the component rather than an absolute scale.

**Supplementary Figure S8.** Effect of age deconfounding on quartile-stratified IDP-cognition coefficients

**Supplementary Figure S8** shows the quartile-stratified IDP-cognition coefficients without age deconfounding. Because brain structure and cognition both vary strongly with age, omitting age terms allows shared age dependence to dominate the coefficients, making patterns appear broadly similar across quartiles. By contrast, when age (and age-related terms) is regressed from both IDPs and cognition, the remaining coefficients better reflect age-dependent differences in the residual brain-cognition association, rather than trivial age-proxy effects.


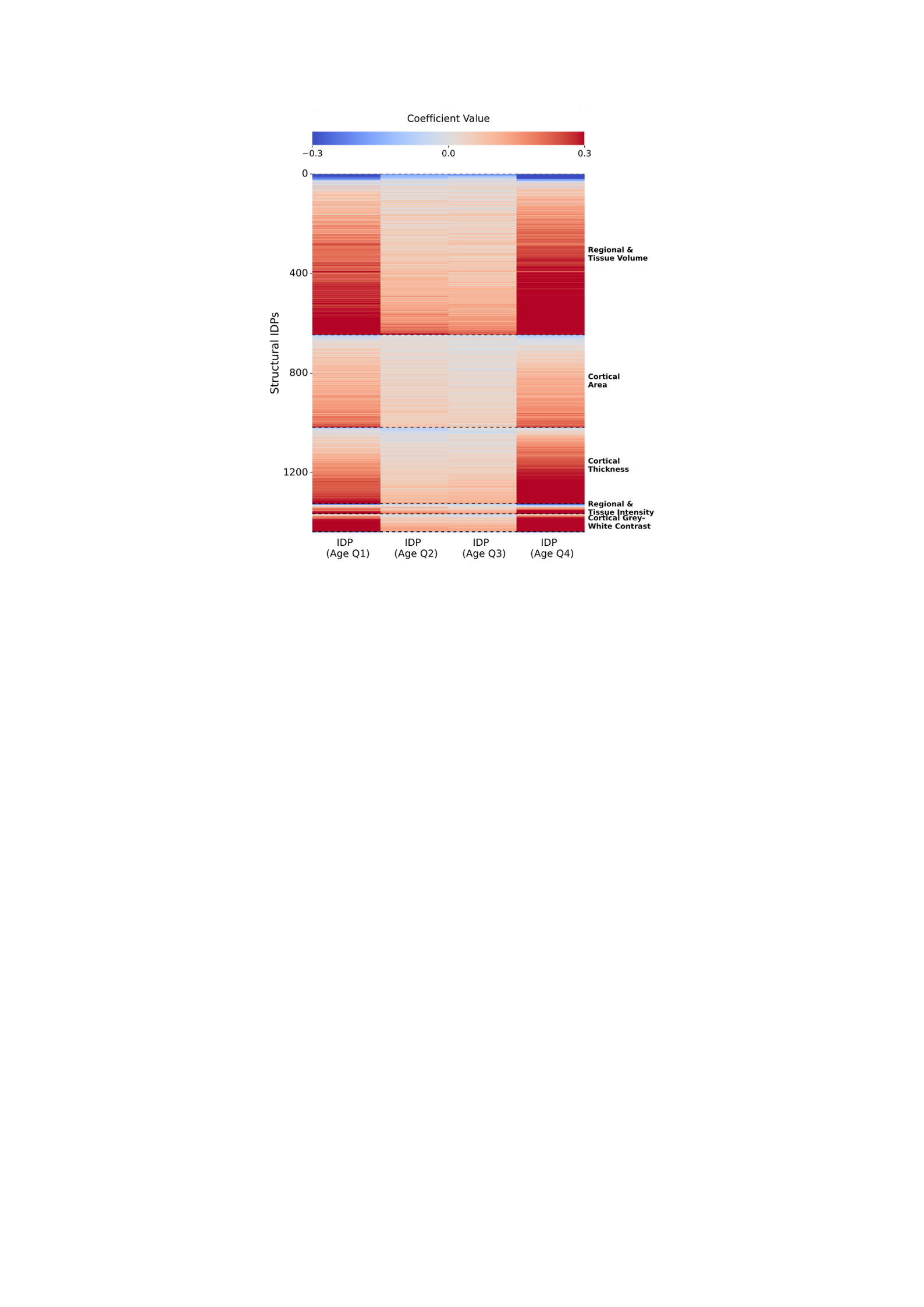

***Figure S8.*** *Effect of omitting age deconfounding on quartile-stratified coefficients*. Heatmap of quartile-specific regression coefficients linking each structural IDP to cognition without regressing out age-related variance. Columns correspond to age quartiles (Q1-Q4); rows show all 1,439 IDPs grouped by category. Compared with the age-deconfounded coefficients (**Figure 2a**), patterns here are dominated by shared age dependence and are therefore broadly similar across quartiles, consistent with structural IDPs acting as proxies for chronological age rather than reflecting age-dependent differences in residual brain-cognition associations.**Supplementary Figure S9.** Age-quartile differences in IDP–cognition associations**
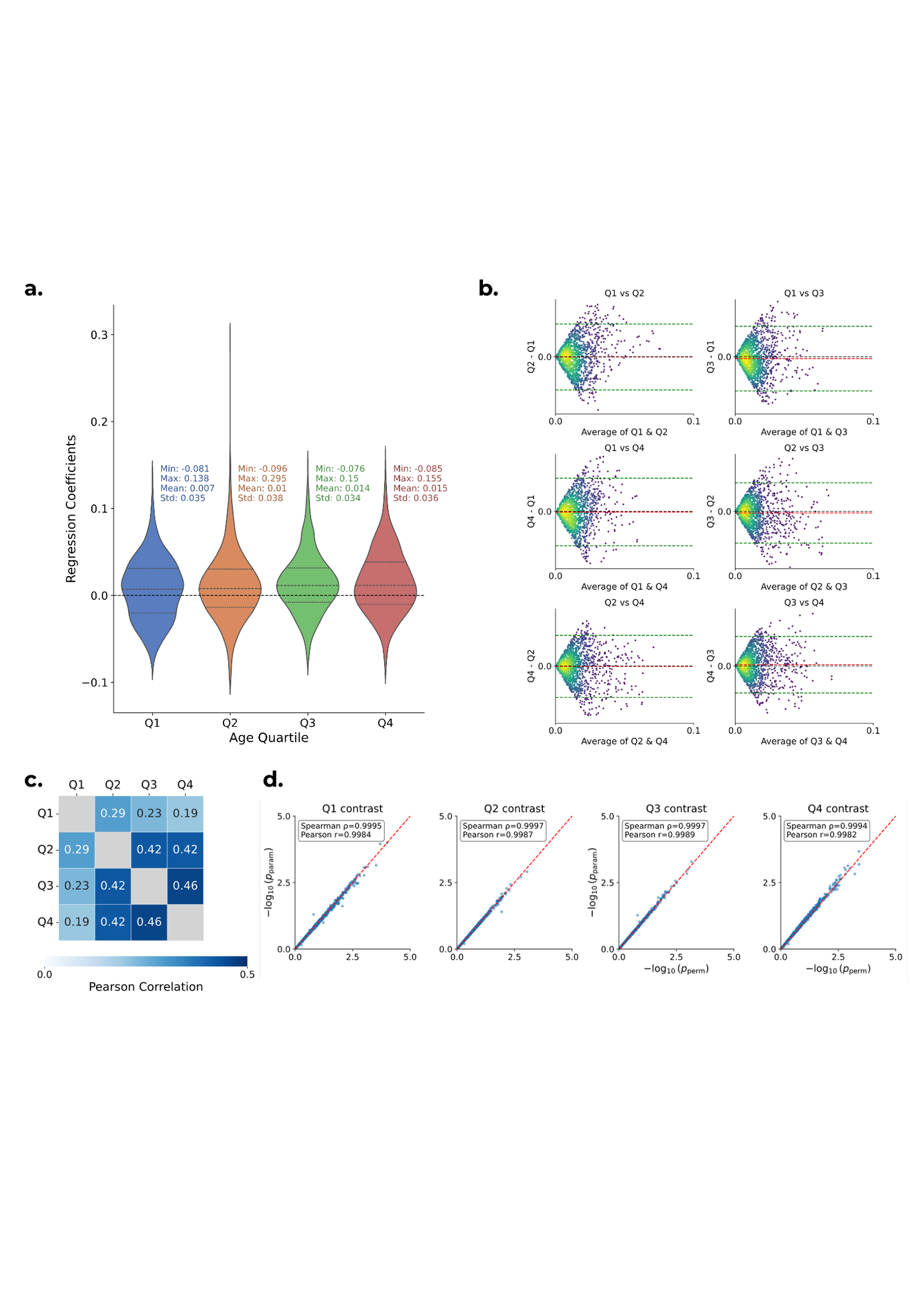
**

**Figure S9.** Summary of how the relationship between structural IDPs and cognition varies across age quartiles. **(a)** Violin plots showing the distribution of regression coefficients across 1,439 IDPs for each age quartile (Q1-Q4), from youngest to oldest. **(b)** Bland-Altman plots comparing Fisher-transformed IDP-cognition correlations across pairs of age quartiles. Each plot shows the difference in correlation (y-axis) against the average correlation (x-axis). Mean differences and 95% limits of agreement are shown with dashed lines. **(c)** Heatmap of Pearson correlations between the absolute values of IDP coefficients across quartiles. Q1 shows particularly low correlation with the other age groups, reinforcing age-specificity in structure-cognition links. **(d)** Parametric vs permutation inference agreement. Scatter plots compare -log_10_ parametric p-values and permutation-based p-values for quartile contrast tests (Q1-Q4 vs the mean of the remaining quartiles) across all structural IDPs. The dashed line indicates the identity line. High Spearman and Pearson correlations across all contrasts indicate near-monotonic agreement between parametric and permutation inference, with deviations confined to the most extreme tail.

**Supplementary Figure S10.** Age-moderation effects between the youngest and oldest age quartiles

**
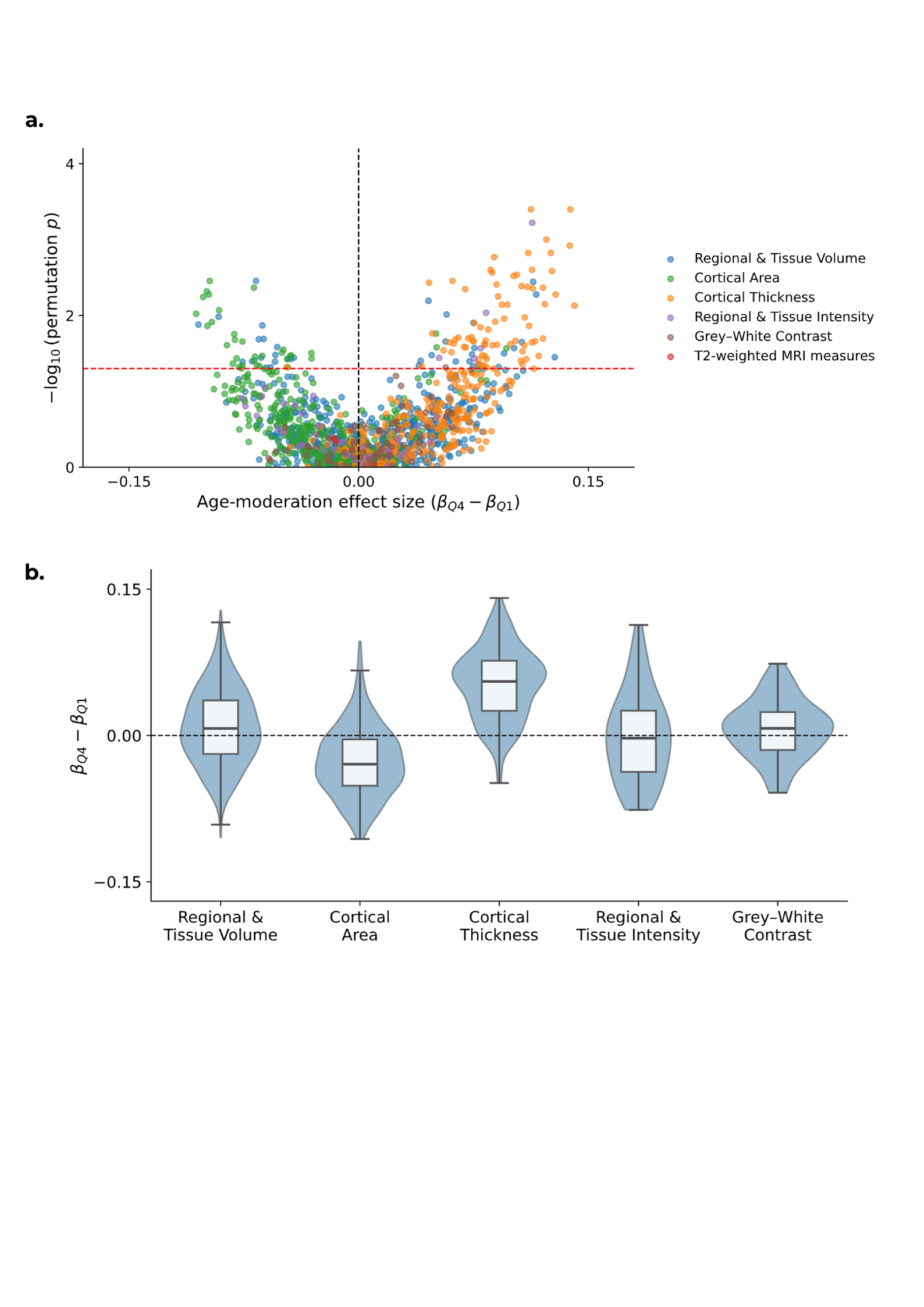
**

**Figure S10.** Age-moderation effects between the youngest and oldest age quartiles across structural IDPs and IDP categories. **(a)** Volcano plot showing age-moderation effect size for each structural IDP, quantified as the difference in regression coefficients between the oldest and youngest age quartiles (β_Q4_ − β_Q1_), plotted against permutation-based significance (−log₁₀ permutation p-value) for the Q4 contrast. Each point corresponds to one IDP and is coloured by IDP category. The vertical dashed line indicates no difference between quartiles, and the horizontal dashed line corresponds to an uncorrected permutation threshold of p = 0.05. This panel illustrates the joint distribution of effect sizes and permutation evidence, highlighting that the strongest age-dependent effects are primarily driven by cortical thickness measures. **(b)** Distribution of age-moderation effect sizes (βQ4 − βQ1) summarised at the category level using violin plots with overlaid boxplots. The dashed horizontal line denotes zero, indicating no difference between age quartiles. Cortical thickness IDPs show a systematic positive shift, whereas cortical area IDPs tend to show weaker or negative shifts, consistent with category-specific patterns of age moderation.

**Supplementary Figure S11.** Model performance expressed using RMSE-based metrics

For transparency, we first report raw RMSE values (**Figure S11a**). As expected, across-age-group models show the largest errors, but pooled training yields slightly lower RMSE than within-age-group training. However, RMSE alone does not account for differences in target variance across test groups, making direct comparisons less meaningful. To address this, we also computed accuracy as percent improvement over a null predictor (%ΔRMSE; **Figure S11b**), which normalises error relative to the baseline variability of each group. Using this variance-adjusted metric, the ranking matches that observed with correlation: within-age-group > pooled > across-age-group. The gap between within and pooled models is small, whereas both clearly outperform across-age-group models. Consistent with modest effect sizes in brain-cognition prediction, absolute improvements were small (< 1%).

**Figure S11c** and **S11d** extend these analyses to the full set of training/testing combinations. Here, raw RMSE values show that the largest errors occur for *Train: Young; Test: Old*, consistent with the greater difficulty of generalising from younger to older subjects. By contrast, Train: Old; Test: Young produces relatively low RMSE despite performing poorly in correlation, likely reflecting differences in variance between test groups. To account for this, we also report %ΔRMSE (**Figure S11d**). This variance-adjusted measure preserves the broad ordering of within-age-group > pooled > across-age-group but highlights that *Train: Young; Test: Old* achieves only modest improvement despite good correlation. This illustrates that Pearson correlation (r) can remain high even when predictions are under-dispersed, whereas RMSE-based metrics penalise this shrinkage, leading to weaker %ΔRMSE gains.


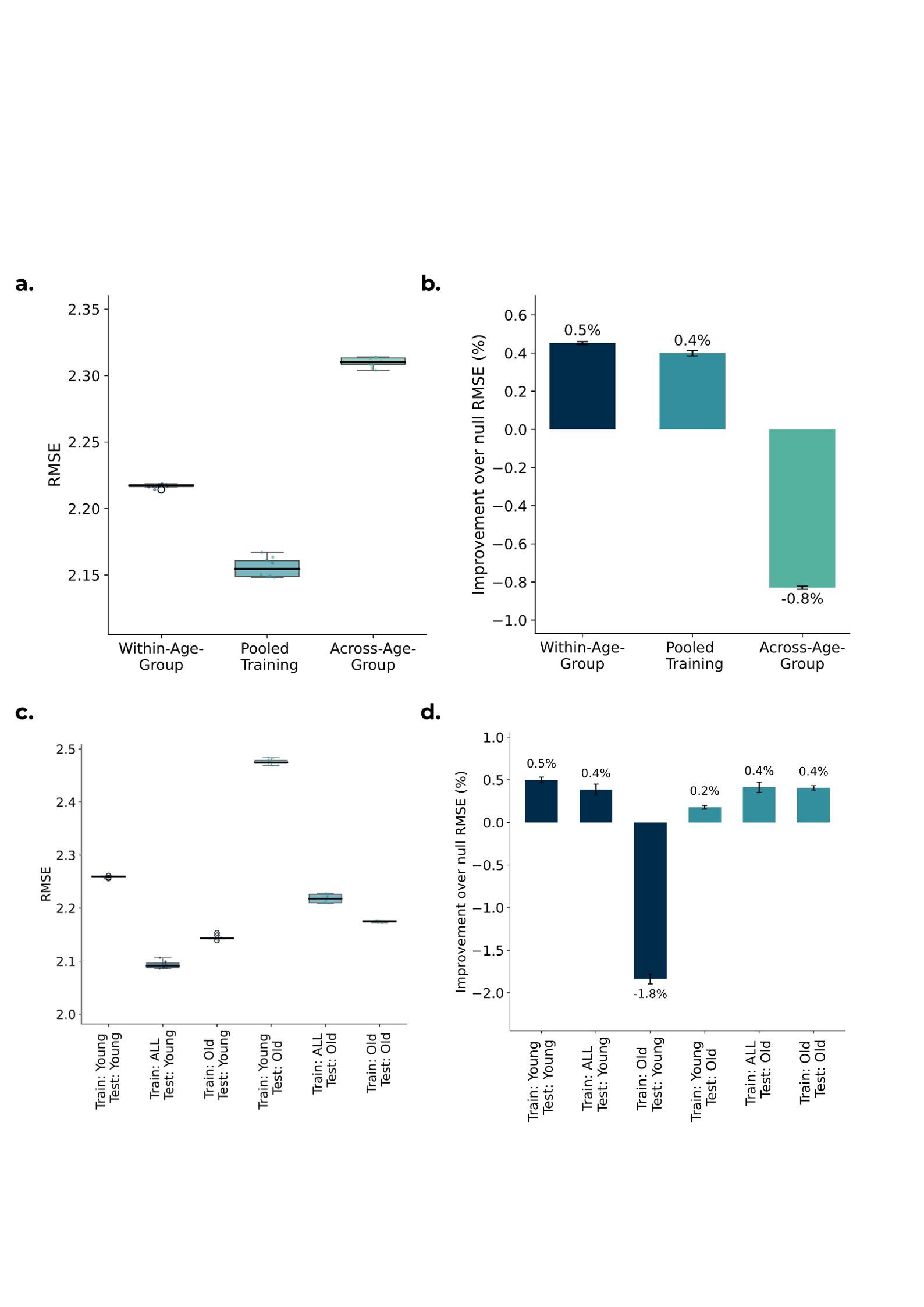


***Figure S11.*** *Model performance expressed using RMSE-based metrics.* **(a)** Raw RMSE values for within-age-group, pooled, and across-age-group models. **(b)** Accuracy expressed as percent improvement over a null predictor (%ΔRMSE), where the null predictor is the mean cognition score in the test set. **(c)** Raw RMSE values for all training/testing combinations. **(d)** Corresponding %ΔRMSE values.

**Supplementary Figure S12.** Paired accuracy differences quantify asymmetry in age generalisation


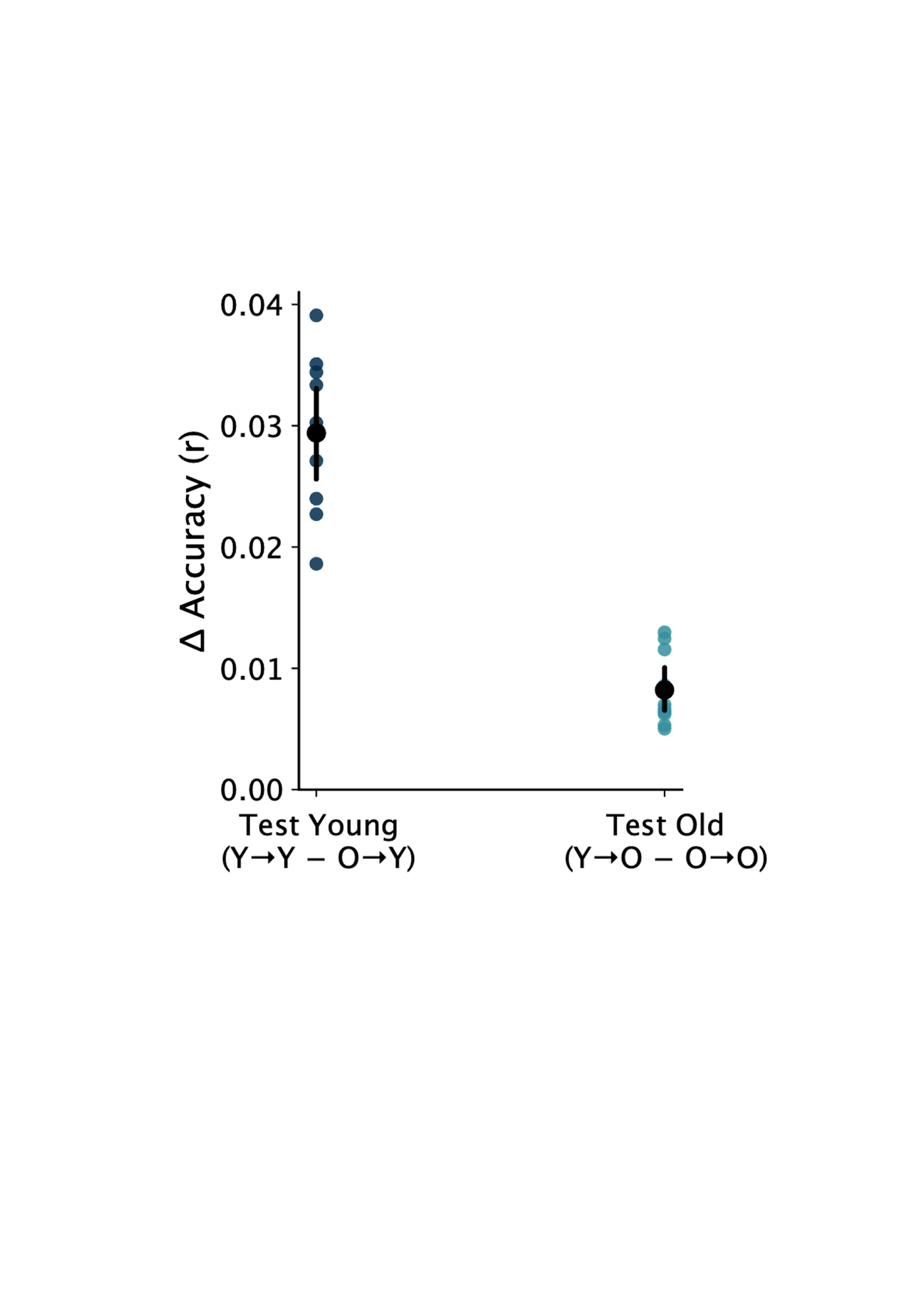


***Figure S12.*** *Paired differences in prediction accuracy (Δr, Pearson correlation) across matched cross-validation repetitions comparing young-trained versus old-trained models within each test group.* **Left:** Test Young contrast (Y→Y − O→Y). **Right:** Test Old contrast (Y→O − O→O). Each point represents one repetition; black markers show the mean paired difference and vertical bars indicate 95% bootstrap confidence intervals across repetitions. Positive values indicate higher accuracy for young-trained models.

**Supplementary Figure S13.** Elastic-net regularisation across training and testing strategies

Elastic net is a widely used approach in neuroimaging prediction studies because it balances shrinkage and sparsity in the presence of many correlated features (Farahibozorg et al., 2021; Pervaiz et al., 2020; Roibu et al., 2023; Shen et al., 2011). We used elastic net here with both α and L1 ratio selected by nested cross-validation. In the age-specific models (Train: Young and Train: Old), the mixing parameter was consistently near zero, indicating ridge-like behaviour. This shows that differences between age-specific training strategies are driven primarily by the strength of regularisation (α), not by a shift toward sparse solutions. Pooled models (Train: ALL) selected slightly higher but still small L1 ratios (0-0.25), which may reflect the presence of features that are predictive in only one age group and are therefore pruned when training on the combined population. In line with prior work, we treat elastic net as a predictive tool rather than an inferential model. Accordingly, we do not interpret individual coefficients but instead focus on broad differences across training strategies and IDP categories.


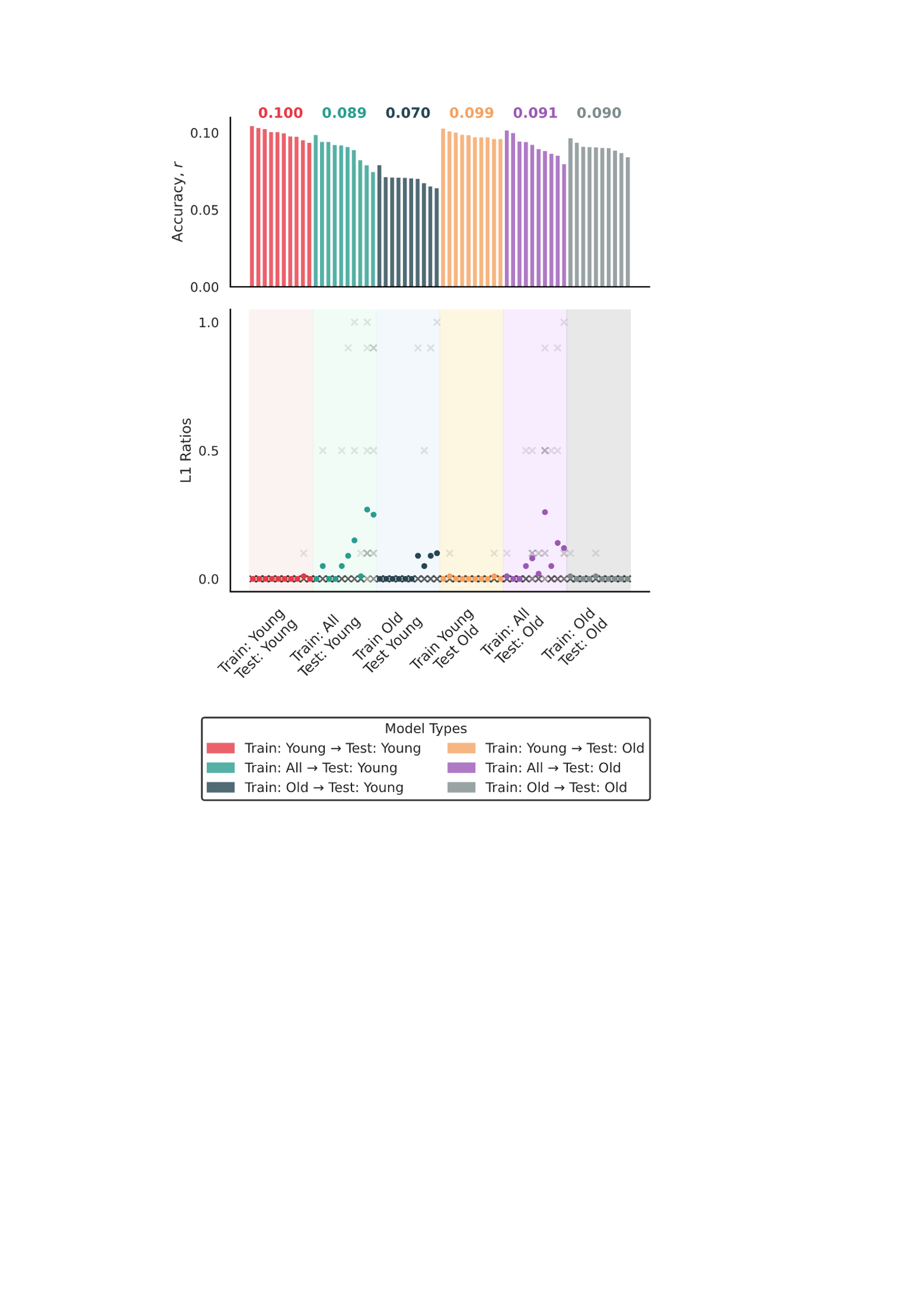


**Figure S13.** Elastic net mixing parameter (L1 ratio) across training strategies. **Top:** Prediction accuracy (r) sorted within each training (numbers above bars indicate the mean r). **Bottom:** L1 ratio selected by nested cross-validation for each repetition (× = individual folds; ● = mean across 10 folds). Age-specific models (Train: Young and Train: Old) clustered near L1 ratio ≈ 0, whereas pooled models (Train: ALL) consistently selected small but non-zero values. Shaded panels indicate the six training and testing conditions.

**Supplementary Tables**

**Table S1.** Summary of confound groups used in the analysis, along with the number of variables in each group (n = 562).

| **Confound no.** | **Confounds Group** | **Number of Confounds** |
| --- | --- | --- |
| 1 | Age | 3 |
| 2 | Sex | 3 |
| 3 | Age by Sex | 3 |
| 4 | Head Size | 3 |
| 5 | Site | 2 |
| 6 | Batch | 16 |
| 7 | Protocol | 7 |
| 8 | Service Pack | 1 |
| 9 | Freesurfer T2 | 3 |
| 10 | Scaling | 9 |
| 11 | Echo Time | 6 |
| 12 | Structural Motion | 3 |
| 13 | Table Position | 12 |
| 14 | Nonlinear Registration | 134 |
| 15 | Crossed Terms | 216 |
| 16 | Acquisition Time | 59 |
| 17 | Acquisition Date | 82 |

**Table S2.** List of the 17 essential confounds used in the primary deconfounding model.

| **Confound no.** | **Essential Confound Name** |
| --- | --- |
| 1 | Age at Site 1 |
| 2 | Age at Site 2 |
| 3 | Age at Site 3 |
| 4 | Age by Sex at Site 1 |
| 5 | Age by Sex at Site 2 |
| 6 | Age by Sex at Site 3 |
| 7 | Sex at Site 1 |
| 8 | Sex at Site 2 |
| 9 | Sex at Site 3 |
| 10 | Site 1 vs Site 2 |
| 11 | Site 1 vs Site 3 |
| 12 | Head Size at Site 1 |
| 13 | Head Size at Site 2 |
| 14 | Head Size at Site 3 |
| 15 | Head Motion at Site 1 |
| 16 | Head Motion at Site 2 |
| 17 | Head Motion at Site 3 |

***Table S3.*** *Variance explained by confounds and incremental age-related variance (ΔR²).* R² values quantify the variance explained in the cognitive composite (PC1) by (i) a non-age confound set and (ii) a full confound set that additionally includes age-related terms (ordinary least squares with an intercept). The incremental age-related contribution is reported as ΔR² = R²(full) − R²(non-age). For structural IDPs, the table summarises the distribution of ΔR² across the 1,439 IDPs (median and selected percentiles). Values are computed on the final analysis sample (N = 25,170; after excluding participants with missing age or any of the 30 cognitive traits); missing IDP values were mean-imputed prior to computing R².

| **Metric** | **Value** |
| --- | --- |
| ***Cognitive composite (PC1)*** |  |
| R² explained by non-age confounds | 0.063 |
| R² explained by full confounds | 0.220 |
| ΔR² (age-related part; full − non-age) | 0.157 |
|  |  |
| ***Structural IDPs: distribution of ΔR² across features*** |  |
| Median ΔR² | 0.018 |
| 25th percentile (p25) ΔR² | 0.006 |
| 75th percentile (p75) ΔR² | 0.044 |
| 95th percentile (p95) ΔR² | 0.112 |
| Maximum ΔR² | 0.269 |

**Table S4.** Distribution of the composite cognitive measure (i.e., the first PC of the top 30 cognitive traits; PC1) across age quartiles. Shown mean cognition score, standard deviation (SD) of cognition scores, and approximate age ranges for each quartile. While means increase systematically with age, SDs remain similar (≈2.1-2.3), indicating that differences in coefficients between quartiles are not driven by unequal outcome variance.

| **Quartile** | **Mean** | **SD** | **Age range** |
| --- | --- | --- | --- |
| Q1 | -1.51 | 2.09 | ≤ 58.3 |
| Q2 | -0.55 | 2.13 | 58.3-64.0 |
| Q3 | 0.49 | 2.24 | 64.0-69.7 |
| Q4 | 1.64 | 2.31 | > 69.7 |

**Table S5.** Top 30 UK Biobank cognitive traits used to construct the composite cognitive measure, with variable names and corresponding Field IDs.

| **Var. no.** | **Column Header** | **UKB Field ID** |
| --- | --- | --- |
| 1 | Duration spent answering each puzzle (2.0) | 6333 |
| 2 | Duration spent answering each puzzle (2.1) | 6333 |
| 3 | Duration spent answering each puzzle (2.4) | 6333 |
| 4 | Duration spent answering each puzzle (2.7) | 6333 |
| 5 | Duration to complete alphanumeric path (trail #2) (2.0) | 20157 |
| 6 | Duration to complete numeric path (trail #1) (2.0) | 20156 |
| 7 | Duration to entering symbol choice (2.11) | 6325 |
| 8 | Duration to entering symbol choice (2.16) | 6325 |
| 9 | Duration to entering symbol choice (2.20) | 6325 |
| 10 | Duration to entering symbol choice (2.22) | 6325 |
| 11 | Duration to entering symbol choice (2.24) | 6325 |
| 12 | Duration to entering symbol choice (2.4) | 6325 |
| 13 | Duration to entering symbol choice (2.8) | 6325 |
| 14 | Duration to first press of snap-button in each round (0.5) | 404 |
| 15 | Duration to first press of snap-button in each round (0.7) | 404 |
| 16 | Duration to first press of snap-button in each round (2.10) | 404 |
| 17 | Duration to first press of snap-button in each round (2.11) | 404 |
| 18 | Duration to first press of snap-button in each round (2.5) | 404 |
| 19 | Duration to first press of snap-button in each round (2.7) | 404 |
| 20 | Fluid intelligence score (2.0) | 20016 |
| 21 | Mean time to correctly identify matches (0.0) | 20023 |
| 22 | Mean time to correctly identify matches (2.0) | 20023 |
| 23 | Number of puzzles attempted (2.0) | 6374 |
| 24 | Number of puzzles correct (2.0) | 20760 |
| 25 | Number of puzzles correctly solved (2.0) | 6373 |
| 26 | Number of symbol digit matches attempted (2.0) | 23323 |
| 27 | Number of symbol digit matches made correctly (2.0) | 23324 |
| 28 | Number of word pairs correctly associated (2.0) | 20197 |
| 29 | Time to complete round (0.2) | 400 |
| 30 | Time to complete round (2.2) | 400 |

Farahibozorg, S. R., Bijsterbosch, J. D., Gong, W., Jbabdi, S., Smith, S. M., Harrison, S. J., & Woolrich, M. W. (2021). Hierarchical modelling of functional brain networks in population and individuals from big fMRI data. *NeuroImage*, *243*. https://doi.org/10.1016/j.neuroimage.2021.118513

Fisher, R. A. (1970). *Statistical Methods for Research Workers* (14th ed.). Oliver & Boyd.

Nichols, T. E., & Holmes, A. P. (2002). Nonparametric permutation tests for functional neuroimaging: A primer with examples. *Human Brain Mapping*, *15*(1). https://doi.org/10.1002/hbm.1058

Pervaiz, U., Vidaurre, D., Woolrich, M. W., & Smith, S. M. (2020). Optimising network modelling methods for fMRI. *NeuroImage*, *211*. https://doi.org/10.1016/j.neuroimage.2020.116604

Roibu, A. C., Adaszewski, S., Schindler, T., Smith, S. M., Namburete, A. I. L., & Lange, F. J. (2023). Brain Ages Derived from Different MRI Modalities are Associated with Distinct Biological Phenotypes. *Proceedings - 2023 10th IEEE Swiss Conference on Data Science, SDS 2023*. https://doi.org/10.1109/SDS57534.2023.00010

Shen, L., Kim, S., Qi, Y., Inlow, M., Swaminathan, S., Nho, K., Wan, J., Risacher, S. L., Shaw, L. M., Trojanowski, J. Q., Weiner, M. W., & Saykin, A. J. (2011). Identifying neuroimaging and proteomic biomarkers for MCI and AD via the elastic net. *Lecture Notes in Computer Science (Including Subseries Lecture Notes in Artificial Intelligence and Lecture Notes in Bioinformatics)*, *7012 LNCS*. https://doi.org/10.1007/978-3-642-24446-9_4

Storey, J. D. (2002). A direct approach to false discovery rates. *Journal of the Royal Statistical Society. Series B: Statistical Methodology*, *64*(3). https://doi.org/10.1111/1467-9868.00346

Storey, J. D., & Tibshirani, R. (2003). Statistical significance for genomewide studies. *Proceedings of the National Academy of Sciences of the United States of America*, *100*(16). https://doi.org/10.1073/pnas.1530509100
